# From pathogenesis to treatment: the role of autophagic cell death in GONFH and its potential mitigation by naringenin

**DOI:** 10.7150/thno.129809

**Published:** 2026-02-18

**Authors:** Huihui Xu, Haipeng Huang, Kaiao Zou, Xingfang Yu, Qinghe Zeng, Congzi Wu, Wenzhe Chen, Pinger Wang, Bangjian He, Luwei Xiao, Jiali Chen, Peijian Tong, Hongting Jin

**Affiliations:** 1 Institute of Orthopaedics and Traumatology of Zhejiang Province, The First Affiliated Hospital of Zhejiang Chinese Medical University (Zhejiang Provincial Hospital of Chinese Medicine), Hangzhou, Zhejiang 310006, China.; 2 The First College of Clinical Medicine, Zhejiang Chinese Medical University, Hangzhou, Zhejiang, 310053, China.

**Keywords:** Apoptosis, autophagy, bone marrow mesenchymal stem cells, ferroptosis, glucocorticoid-associated osteonecrosis of the femoral head, naringenin

## Abstract

**Rationale:**

Glucocorticoid (GC)-associated osteonecrosis of the femoral head (GONFH) is an incurable orthopedic illness. Reduced osteogenic differentiation of bone marrow mesenchymal stem cells (BMSCs) is at the core of the pathogenesis of GONFH; however, its molecular mechanism remains unclear. The study aimed to explore the pathological mechanisms of GONFH and to investigate the efficacy and mechanism of naringenin (NAR) in treating GONFH.

**Methods:**

RNA sequencing was conducted to investigate the pathogenesis of GONFH and identify the potential therapeutic mechanism of NAR. The levels of autophagy, ferroptosis, apoptosis, and osteogenesis were examined in clinical, animal, and BMSC samples. Moreover, the specific binding of NAR to ULK1 and its role in promoting ser757 phosphorylation of ULK1, leading to reduced autophagy-dependent cell death and increased osteogenic differentiation of BMSCs, were investigated using molecular dynamics simulations and systematic in vivo and in vitro experiments.

**Results:**

In clinical, animal, and BMSCs samples, autophagy, ferroptosis, and apoptosis were notably increased in the GONFH group, while osteogenesis was markedly decreased. In addition, the effects of rapamycin (RAPA, an autophagy agonist) and 3-methyladenine (3-MA, an autophagy inhibitor) were investigated to confirm that the GC-induced decrease in osteogenic differentiation of BMSCs is mediated through autophagy-dependent cell death. Additionally, NAR exhibits high affinity for ULK1, which increases its inhibitory phosphorylation at ser757. This particular communication inhibits GC-induced autophagy and subsequent cell death, thereby normalizing osteogenic differentiation of BMSCs. It is interesting to note that the protective effects of NAR were abolished by pharmacological (RAPA) and genetic (ULK1-S757A mutation) interventions.

**Conclusions:**

Taken together, our work elucidates a pathogenic process involving autophagy-dependent cell death and defines NAR as a specific treatment that regulates ULK1 to halt this pathogenic cascade.

## Introduction

Osteonecrosis of the femoral head (ONFH) is an incurable, eventually locally dysfunctional, orthopedic disease that causes severe impairment of hip pain and functionality. The most common cause of non-traumatic ONFH is glucocorticoid (GC), with the burden of annual ONFH incidence being enormous on the planet 1, 2. Although it is common, the pathogenesis of GC-associated ONFH (GONFH) and its effective treatment using drugs are among the most significant clinical issues. That is why it is essential to thoroughly understand the pathogenesis of GONFH to develop effective therapeutic agents.

One of the primary factors in the pathogenesis and evolution of GONFH is the GC-induced reduction in the osteogenic differentiation of bone marrow mesenchymal stem cells (BMSCs) 3, 4. BMSCs possess multiple differentiation potentials, enabling osteogenic, chondrogenic, and adipogenic differentiation, thereby playing a crucial role in skeletal development 5. Nevertheless, due to the exposure to supraphysiological levels of GC, BMSCs also become vulnerable to different types of regulatory cell death (RCD). Although the role of apoptosis induced by GC has been well-established, its mechanism of action remains unclear 6. Recently, ferroptosis, an iron-dependent form of cell death caused by lipid peroxidation and the inactivation of glutathione peroxidase 4 (GPX4), has also been identified as a contributor to GONFH pathogenesis 7. Recent studies have demonstrated that ferroptosis, in addition to apoptosis, can mediate the inhibitory effect of GC on osteogenic differentiation, with implications for clinical interventions 8-10.

Autophagy exerts a double-edged sword effect in BMSCs, ranging from cellular protection under oxidative stress to inducing cell death through sustained activation 11, 12. Autophagy also plays a key role as a control node that integrates RCD (mainly ferroptosis and apoptosis) 13-15. Although autophagy-dependent regulation has been demonstrated in various pathologies, such as cancer, organ fibrosis, and osteoporosis, the connection between autophagy-dependent regulation and GONFH pathogenesis remains to be established.

Herbal medicines have provided new avenues of therapy for orthopedic diseases due to their good efficacy and safety profile 16, with several compounds demonstrating therapeutic potential for GONFH 17-20. Naringin (NAR), a flavonoid compound found in citrus fruits, exhibits anti-inflammatory and anti-cancer properties due to its low toxicity, making it a potential therapeutic agent 21-24. Relevant to orthopedics, NAR can enhance bone formation, as evidenced in osteoporosis models 25. However, its ability to treat GONFH and the mechanism involved are completely unexplored. This study aims to fill this gap by defining the pathogenesis of GONFH and systematically investigating the therapeutic efficacy and mechanism of NAR.

## Materials and Methods

### Human femoral head samples

Human femoral head samples were acquired from patients with advanced GONFH (15 in total) or femoral neck fracture (FNF, 10 in total) who underwent total hip arthroplasty at the Department of Orthopedics, The First Affiliated Hospital of Zhejiang Chinese Medical University. The research was permitted by the Ethics Committee of the First Affiliated Hospital of Zhejiang Chinese Medical University (Permit Number: 2018-KL-005-02).

### Rat BMSCs isolation, culture and treatment

BMSCs isolation was conducted as previously described 3. The third passage BMSCs were incubated in α-modified Eagle's medium (α-MEM, 6125602, Gibco) containing 10% fetal bovine serum (FBS, SA201.02, CellMax) in a 5% CO_2_ humidified incubator at 37 °C. BMSCs between different groups were incubated with the autophagy agonist-rapamycin (RAPA, 50 nM 26, HY-10219, MedChemExpress), autophagy inhibitor-3-methyladenine (3-MA, 2 mM 27, HY-19312, MedChemExpress), ULK1 ser757 mutant lentivirus (Shanghai Genechem Co., Ltd., Shanghai, China), and NAR (5, 10, and 20 μM; S2394, Selleck), respectively, before administration of dexamethasone (DEX, S1322, Selleck).

### RNA sequencing

The total RNA of FNF patients and GONFH patients (n = 5/group) and BMSCs from the control (CTRL) group, DEX-treated group, and NAR-treated group (n = 3/group) were extracted using a RNeasy Micro Kit (QIAGEN) with TRIzol reagent (Invitrogen, Carlsbad). The concentration and quality of RNAs were assessed by an Agilent 2100 Bioanalyzer (Agilent Technologies) and a Nanodrop 2000 (Thermo, Waltham). The mRNA-seq libraries were prepared using the VAHTS TM mRNA-seq V2 Library Prep Kit for Illumina (Vazyme, #NR601) and then run on an Illumina HiSeq X-Ten next-generation sequencer. FastQC (version 0.11.9) was used for quality control and read trimming. Transcripts expressed at or above 1 count per million reads in our samples were selected for analysis. Differentially expressed genes (DEGs) were identified by a linear regression model package. Kyoto encyclopedia of genes and genomes (KEGG) enrichment analysis of DEGs was conducted using the clusterProfiler R package.

### Animal studies

Seventy 16-week-old male Sprague-Dawley rats (weight: 550 ± 50 g) were obtained from the Experimental Animal Research Center of Zhejiang Chinese Medical University. GONFH rat models were established by tail vein administration of lipopolysaccharide (LPS, 200 μg/kg; L6011, Sigma-Aldrich) combined with intraperitoneal administration of methylprednisolone (MPS, 100 mg/kg; EL4280, Pfizer), as previously described 19, 20, 28, 29. Seventy rats were randomized into 7 groups, as CTRL group; GONFH group; NAR low, medium and high dose treatment groups (rats in the GONFH group received NAR 5, 10 and 20 mg/kg/day orally for 6 weeks 30 respectively); NAR + RAPA treatment group (rats in the GONFH group received NAR 20 mg/kg/day orally combined with RAPA 2 mg/kg/day, 3 days/week, intraperitoneally for 6 weeks) 31 and alendronate (ALN, U026764, Rovi Pharma Industrial Services S.A) treatment group (as positive control) (rats in the GONFH group received ALN 3 mg/kg/week orally for 6 weeks 32. The current study was permitted by the Ethics Committee of Zhejiang Chinese Medical University (Permit Number: 202006-0244).

### Biomechanical testing

The axial compression testing machine (Bose Corp, Minnesota) was employed to apply a static load to the femoral head at a speed of 1 mm/min. The presence of the first mechanical turning point will be determined as the load-bearing capacity of the rat femoral head.

### Molecular docking and dynamics simulation

The structures of AMPK, MTOR, and ULK1 were downloaded from the RCSB PDB, and NAR was obtained from PubChem. After preparation with MGLTools and conversion to PDBQT format, global docking was performed with AutoDock Vina. The highest-affinity complex was selected for molecular dynamics simulations using the GROMACS software. The protein and ligand were parameterized with the Amber14SB and GAFF force fields, respectively, and solvated in a TIP3P water box. The system was minimized, equilibrated under NVT (100 ps) and NPT (100 ps) ensembles, and subjected to a 100 ns production molecular dynamics simulation. Simulations employed a 2 fs time step, PME for electrostatics, and saved frames every 10 ps for analysis.

### Cellular thermal shift assay (CETSA)

CETSA was conducted as previously described 33. Briefly, the BMSCs were divided into NAR and DMSO groups, and the BMSCs were treated with 20 μM NAR or 0.1% DMSO for 12 h, respectively. Subsequently, the BMSCs were divided into aliquots and heated at the specified temperature conditions (32 - 67 °C) for 3 min, followed by three freeze-thaw cycles with liquid nitrogen. The lysate was centrifuged at 20,000 × g for 20 min at 4 °C, and the supernatant was collected for western blotting (WB) analysis.

### *μ*-CT analysis

Human and rat femoral head samples were scanned by an 18 μm resolution *μ*-CT scanner (1176, SkyScan). Image reconstruction and analysis were performed using NRecon v1.8 and CTAn v1.15 software, and parameters analyzed included bone mineral density (BMD), trabecular bone number (Tb.N), trabecular bone volume fraction (BV/TV), trabecular thickness (Tb.Th), and trabecular bone separation (Tb.Sp). In addition, 10 consecutive sagittal images of the region of interest (ROI) were reconstructed in three dimensions using CTVolx v3.0 software, with the subchondral bone of the femoral head as the ROI.

### Histology

After decalcification and dehydration, femoral head samples were embedded in paraffin, cut into 3-μm-thick sections, and subjected to morphological analysis using Alcian Blue Hematoxylin/Orange G (ABH/OG) staining. Histopathologic alterations were evaluated by light microscopy (Axioscope A1; Zeiss), and the proportions of empty bone lacunae, adipocyte area, and bone trabeculae area were measured.

### Immunohistochemistry (IHC) and immunofluorescence (IF) staining

For IHC, femoral head sections were immersed in a sodium citrate solution and heated at 60 °C for four hours to facilitate antigen retrieval. Afterwards, the slices were cultured overnight at 4 °C with the corresponding primary antibodies (Table S1). On day 2, after reacting with the secondary antibody for 20 min, the sections were visualized using a diaminobenzidine solution and counterstained with hematoxylin. For IF, femoral head sections were cultured with fluorescent secondary antibodies for 20 min and incubated with 4',6-diamidino-2-phenylindole (DAPI) for 3 min.

### Cell counting kit-8 (CCK-8) assay

The dosages of DEX (1, 10, 100, and 1000 nM) and NAR (5, 10, 20, and 40 μM) were based on previous studies 9, 34. Specifically, BMSCs were incubated in 96-well plates at a density of 6,000 cells per well in 100 μL of medium containing DEX or NAR. To assay the proliferative activity of BMSCs, 10 μL of CCK-8 solution (C0037, Beyotime) was added to the wells and subsequently incubated for an additional 30 min at 37 °C. A microplate reader (Synergy H1M, BioTek) was employed to determine the absorbance of each well at 450 nm.

### Reactive oxygen species (ROS) detection

To assess the level of ROS in tissue sections, freshly isolated femoral head samples were quickly frozen and cut into sections of 10 μm thickness at a temperature of -20 °C, and ROS levels were determined according to the instructions of the dihydroethidium (DHE)-ROS assay kit (BB-470516, BestBio). Briefly, sections were incubated with DHE for 45 min without light and then incubated with DAPI for 5 min. For intracellular ROS detection, following medication administration, BMSCs cultured on glass coverslips were incubated in a serum-free medium containing 10 μM fluorescent dye 2',7'-dichlorofluorescein diacetate (DCFH-DA; S0033S, Beyotime) for 30 min in darkness.

### Apoptosis assay

To assess the level of apoptosis in tissue sections, we conducted the Terminal Deoxynucleotidyl Transferase-Mediated Nick-End Labeling (TUNEL) assay (C1088, Beyotime) according to the manufacturer's guidelines. Briefly, BMSCs were fixed in 4% paraformaldehyde for 40 min, followed by incubation with 0.3% Triton X-100 for 20 min. BMSCs were then incubated with the TUNEL detection solution without light for 1 h, and then incubated with DAPI for 3 min. For intracellular Annexin V-FITC/PI detection, BMSCs were seeded in 6-well plates and analyzed according to the Annexin V-FITC/PI kit (FXP018, 4A Biotechnology) protocol. The percentage of apoptotic BMSCs was measured using a flow cytometer (CytoFlex S, Beckman Coulter).

### Ad-mCherry-GFP-LC3B adenovirus transfection

Adenovirus Ad-mCherry-GFP-LC3B was acquired from Beyotime (C3011) to assess autophagy flux. Briefly, BMSCs were cultured in 12-well cell culture plates and transfected with Ad-mCherry-GFP-LC3B adenovirus, infected at 40% confluence at a multiplicity of infection of 50. The cells were subjected to the appropriate pharmacological interventions 2 days later. The expression of autophagic flux was observed using a confocal laser scanning microscope (LSM880, Zeiss).

### Transmission electron microscopy (TEM)

BMSCs in each group were immobilized in 2.5% glutaraldehyde at 4 °C overnight, followed by 1% osmium tetroxide fixation for 1 h. Subsequently, the immobilized cells were fixed in 10% gelatin, followed by fixation with glutaraldehyde at 4 °C for another 1 h. Following gradient dehydration with ethanol (30%, 50%, 70%, 90%, 95%, 100%, 100%), the cells were immersed in epoxy resin. They were then sectioned into ultrathin portions using a Leica UC6 (EM UC6, Leica) and visualized with a TEM (JEM1011).

### Intracellular lipid peroxide and ferrous iron assessments

BMSCs were placed in a medium with 5 μM C11 BODIPY (L267, Dojindo) or 1 μM FerroOrange (F374, Dojindo) for 30 min in the dark after drug administration. The stained BMSCs were then observed using fluorescence microscopy (X-Cite 120Q, Lumen Dynamics).

### Superoxide dismutase (SOD), glutathione (GSH) and malondialdehyde (MDA) measurements

The degree of lipid peroxidation and the level of antioxidant system activation in BMSCs were determined using SOD (S0101S, Beyotime), GSH (S0052, Beyotime), and MDA (S0131S, Beyotime) kits, according to the manufacturer's guidelines.

### Osteogenic differentiation assay

BMSCs in different treatment groups were plated in 24-well plates and induced for 14 days in osteoblast induction medium (α-MEM supplemented with 50 µg/ml ascorbic acid and 10 mM β-glycerophosphate). Subsequently, alkaline phosphatase (ALP) staining was performed using the 1-Step NBT/BCIP Kit (Thermo Fisher Scientific, Waltham).

### WB

Total cellular proteins from each group of BMSCs were extracted with a modified radioimmunoprecipitation assay buffer containing a protease inhibitor cocktail on ice for 30 min. Then, 40 μg of targeted protein was loaded and electrophoresed on a sodium dodecyl sulfate-polyacrylamide gel electrophoresis gel and transferred to a polyvinylidene difluoride membrane. The membranes were blocked with 5% skim milk at room temperature for 2 h and then incubated for 12 h at 4 °C with the corresponding primary antibodies (Table S1). Following incubation with the respective fluorescent secondary antibody for 2 h at 4 °C, the blot was visualized with an LI-COR Odyssey scanner (LI-COR Biosciences).

### Reverse transcription-quantitative PCR (RT-qPCR)

Total RNA was obtained utilizing TRIzol® Reagent (Invitrogen), and cDNA was synthesized utilizing PrimeScript™ RT Reagent Kit (Takara). The RT-qPCR was conducted by SYBR Premix EX Taq™ Kit (Takara) following the manufacturer's instructions. The specific primer sequences are shown in Table S2.

### Statistical analysis

All data in this study were expressed as mean ± standard deviation, and statistical analysis was conducted using GraphPad Prism software. Two-tailed unpaired parametric Student's t-test and one-way ANOVA followed by Dunnett's test were performed for statistical analyses. *P* < 0.05 was statistically significant, with *P < 0.05, **P < 0.01, and ***P < 0.001.

## Results

### Excessive GC aggravates BMSCs' ferroptosis and apoptosis while suppressing osteogenesis in GONFH

**Figure 1:**
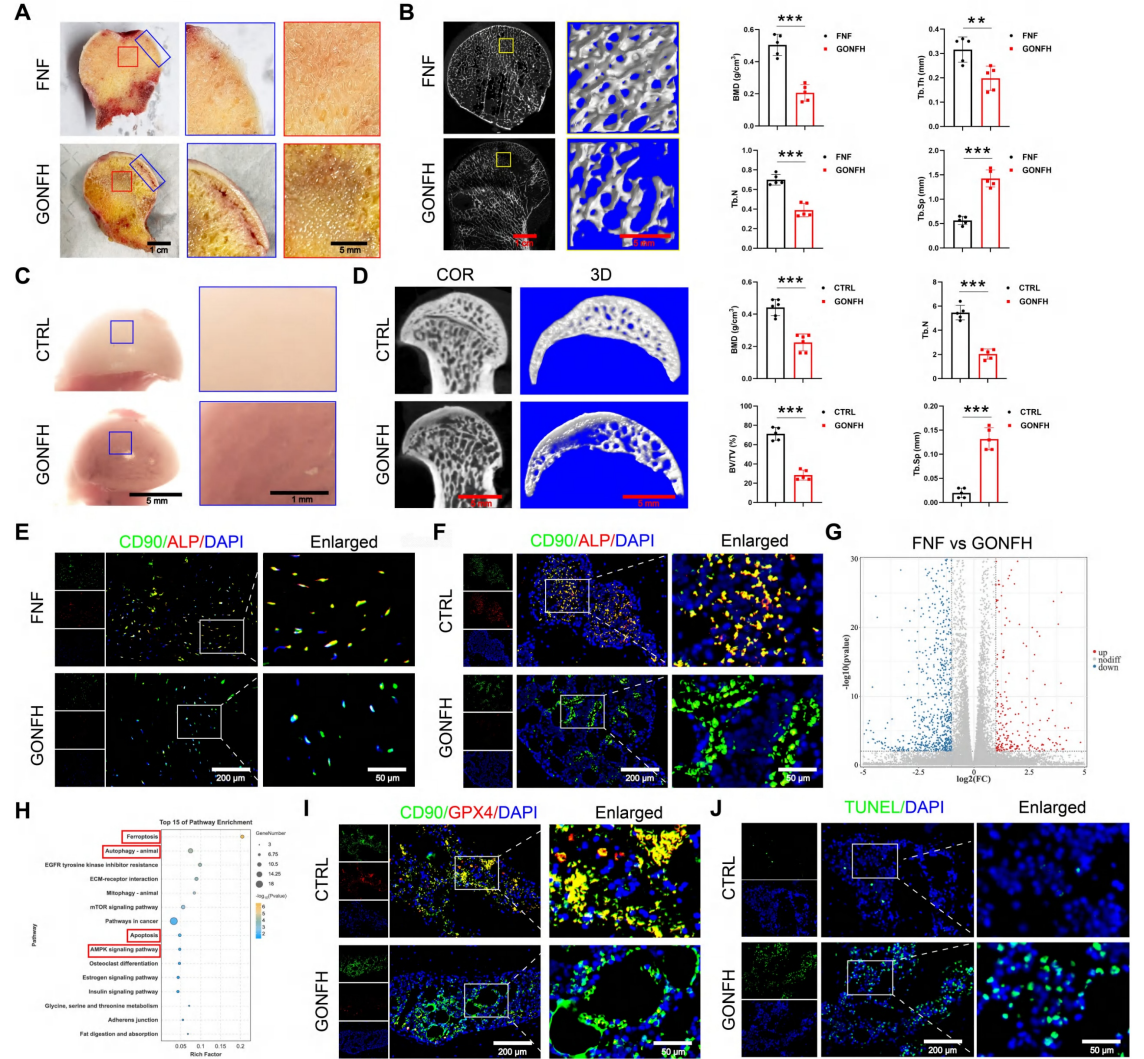
Excess GC exacerbates cellular ferroptosis and apoptosis while reducing bone mass in GONFH patients and rats. (**A**) Gross morphology of human femoral head samples. Blue box indicates areas of necrotic collapse, and red box indicates areas of cystic change. (**B**) Representative *μ*-CT scanning images and quantitative analysis of BMD, Tb.Th, Tb.N and Tb.Sp of human femoral head samples (n = 5). (**C**) Gross morphology of rat femoral head samples. (**D**) Representative *μ*-CT scanning images and quantitative analysis of BMD, BV/TV, Tb.N and Tb.Sp of rat femoral head samples (n = 5). (**E-F**) Representative IF co-staining images of CD90 and ALP of human and rat femoral head samples. (**G**) Volcano diagram of RNA sequencing. (**H**) KEGG enrichment analysis plot of RNA sequencing. (**I**) Representative IF co-staining images of CD90 and GPX4 of rat femoral head samples. (**J**) Representative TUNEL staining images of rat femoral head samples. Data are presented as mean ± SD. **P < 0.01 and ***P < 0.001 (vs. GONFH).

We first characterized the pathological phenotype of GONFH using clinical samples and a rat model. The femoral heads of GONFH patients exhibited hallmark pathological features of subchondral cystic degeneration (red box) and necrotic collapse (blue box) (Figure 1A). *μ*-CT analysis revealed a significant deterioration of bone microstructure in GONFH, characterized by decreased BMD, Tb.N, Tb.Th and BV/TV, alongside an increase in Tb.Sp in both human and rat specimens (Figure 1B, D). GONFH rats also exhibited an abnormal dark red appearance of the femoral head compared to CTRL (Figure 1C). Unexpectedly, CD90 (BMSCs marker) and ALP (osteogenic marker) co-staining showed that the level of osteogenic differentiation of BMSCs was notably reduced in GONFH patients and rats (Figure 1E-F).

To uncover the driving mechanisms behind this phenotype, we conducted RNA sequencing, which revealed extensive transcriptomic alterations in GONFH, with 4,574 genes upregulated and 6,586 genes downregulated (Figure 1G). KEGG analysis revealed enrichment of ferroptosis and apoptosis pathways (Figure 1H). Our findings confirmed increased ferroptosis and apoptosis within the femoral heads of GONFH patients and rats (Figure 1I-J, S1).

**Figure 2:**
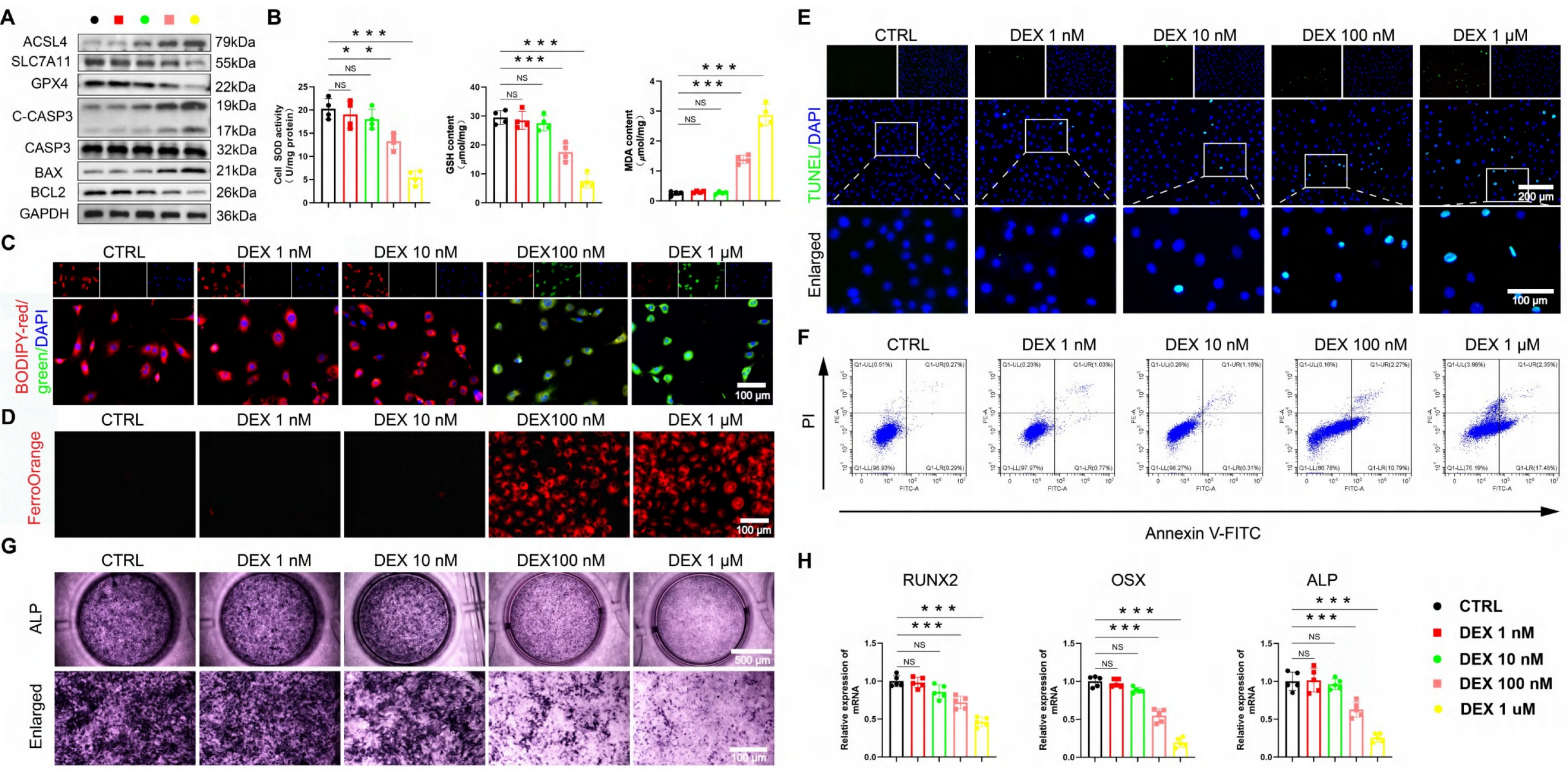
The excessive GC exacerbates ferroptosis and apoptosis, while reducing osteogenic differentiation in BMSCs. (**A**) Protein levels of GPX4, SLC7A11, ASCL4, C-CASP3, CASP3, BAX and BCL2 in BMSCs treated with different concentrations of DEX (n = 3). (**B**) SOD, GSH and MDA levels of BMSCs treated with different concentrations of DEX (n = 4). (**C-D**) C11-BODIPY and FerroOrange staining images of BMSCs treated with different concentrations of DEX. (**E-F**) Representative images of TUNEL staining and Annexin V-FITC/PI assay in BMSCs treated with different concentrations of DEX. (**G**) ALP staining images of BMSCs treated with different concentrations of DEX for 14 days. (**H**) mRNA levels of RUNX2, OSX and ALP in BMSCs treated with different concentrations of DEX (n = 5). Data are presented as mean ± SD. NS no significance, **P < 0.01, and ***P < 0.001 (vs. CTRL).

We employed an established DEX in vitro model 9, 35 to further investigate the above findings. A dose-gradient experiment demonstrated that high-dose DEX (≥ 100 nM) was critical for inducing the dysfunction of BMSCs. DEX dose-dependently elevated ROS, creating a pro-oxidative milieu (Figure S2A-B). Accompanying the marked induction of ferroptosis at high doses, characterized by upregulated acyl-CoA synthetase long-chain family member 4 (ACSL4), downregulated solute carrier family 7 member 11 (SLC7A11) and GPX4, increased MDA, Fe²⁺ and lipid peroxidation, and decreased GSH/SOD (Figure 2A-D, Figure S2C). Apoptosis was also preferentially activated at high DEX concentrations, as shown by elevated cleaved cysteinyl aspartate-specific proteinase 3 (C-CASP3), B-cell lymphoma 2 (BCL2)-associated X protein (BAX)/BCL2 ratio, and positive TUNEL/Annexin V staining (Figure 2A, E-F, S2D-E).

Concomitantly, osteogenic differentiation was robustly suppressed at these same concentrations, with ALP activity and the expression of runt-related transcription factor 2 (RUNX2), osterix (OSX), and ALP being prominently reduced (Figure 2G-H). These data unequivocally link high-level GC exposure to the coordinated induction of ferroptosis and apoptosis, as well as the parallel suppression of osteogenesis in BMSCs, thereby pinpointing a central mechanism for GONFH pathogenesis.

### The AMPK/MTOR/ULK1 signaling pathway mediates GC-induced elevated autophagy in BMSCs

**Figure 3:**
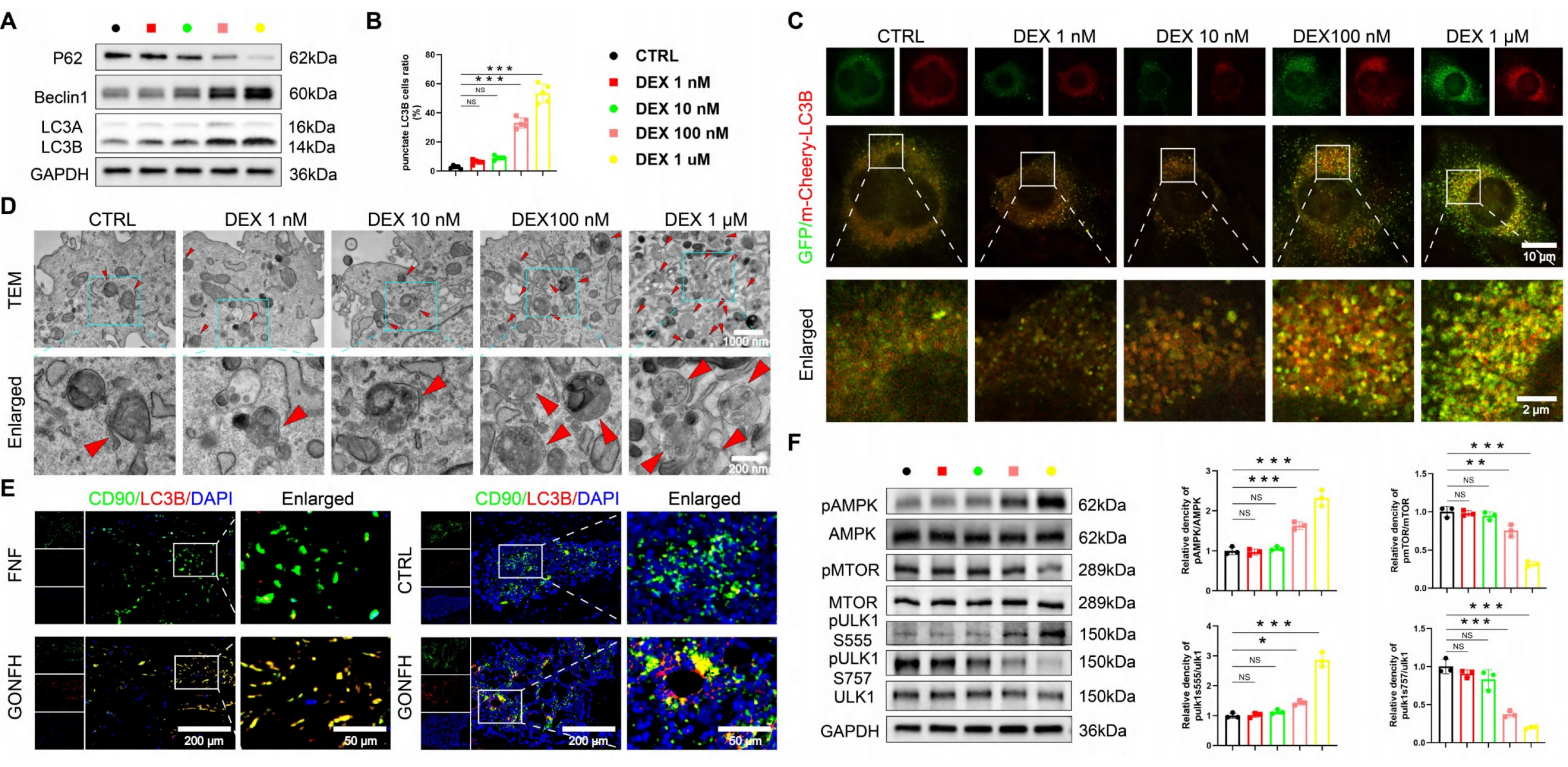
Excess GC leads to excessive autophagy in BMSCs through activation of the AMPK/MTOR/ULK1 signaling pathway. (**A**) Protein levels of P62, Beclin1 and LC3B in BMSCs treated with different concentrations of DEX (n = 3). (**B-C**) Representative images and quantitative analysis of mCherry-GFP-LC3B in BMSCs treated with different concentrations of DEX (n = 5). (**D**) Representative TEM images of BMSCs treated with different concentrations of DEX. (**E**) Representative IF co-staining images of CD90 and LC3B of human and rat femoral head samples. (**F**) Protein levels of AMPK/MTOR/ULK1 signaling pathway-related proteins in BMSCs treated with different concentrations of DEX (n = 3). Data are presented as mean ± SD. NS no significance, **P < 0.01, and ***P < 0.001 (vs. CTRL).

RNA sequencing results indicate autophagy as a pivotal pathway (Figure 1H). We found that DEX induces autophagy in a dose-dependent manner in BMSCs. High concentrations (≥ 100 nM) upregulated autophagic activity, as evidenced by WB analysis of LC3B, Beclin1, and p62, increased LC3B puncta formation, and elevated autophagosome numbers, whereas low doses (≤ 10 nM) were ineffective (Figure 3A-D, S2F). Furthermore, BMSCs from GONFH patients and rats exhibited elevated levels of LC3B (an autophagy marker)/CD90 (a marker for BMSCs) (Figure 3E).

We next sought to identify the upstream signaling mechanism. The AMPK/MTOR/ULK1 pathway, a master regulator of autophagy 36, 37 and a hit in our KEGG analysis (Figure 1H), was a prime candidate. Strikingly, high-dose DEX specifically activated this pathway, shifting the phosphorylation balance towards activation (increased pAMPK and pULK1 ser555) and away from inhibition (decreased pMTOR and pULK1 ser757) (Figure 3F). These data establish a mechanistic link between GC excess, activation of the AMPK/MTOR/ULK1 pathway, and subsequent autophagic hyperactivation in BMSCs.

### GC-impaired osteogenesis is mediated by autophagy-dependent ferroptosis and apoptosis

**Figure 4:**
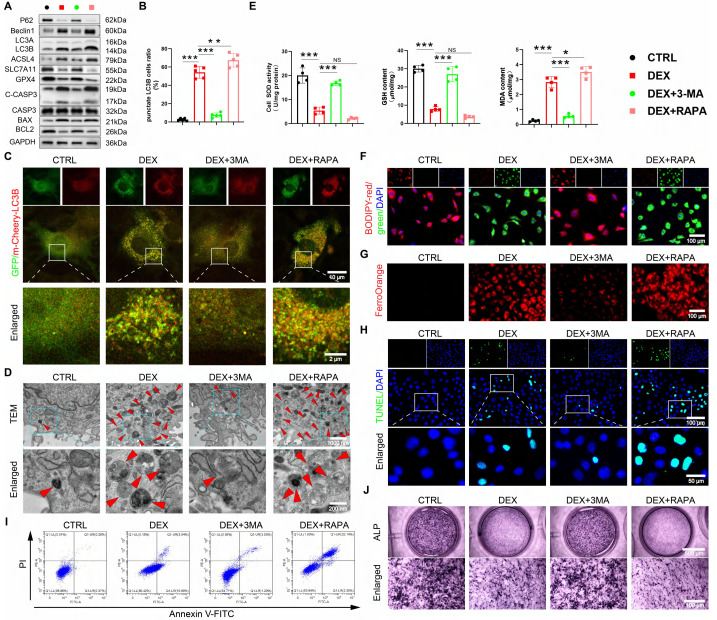
The excessive GC reduces osteogenic differentiation of BMSCs through autophagy-dependent ferroptosis and apoptosis. (**A**) Protein levels of P62, Beclin1, LC3B, GPX4, SLC7A11, ASCL4, C-CASP3, CASP3, BAX and BCL2 in 1 μM DEX, 3-MA, or RAPA-treated BMSCs (n = 3). (**B-C**) Representative images and quantitative analysis of mCherry-GFP-LC3B in 1 μM DEX, 3-MA, or RAPA-treated BMSCs (n = 5). (**D**) Representative TEM images in 1 μM DEX, 3-MA, or RAPA-treated BMSCs. (**E**) MDA, GSH and SOD levels in 1 μM DEX, 3-MA, or RAPA-treated BMSCs (n = 4). (**F-G**) C11-BODIPY and FerroOrange staining images in 1 μM DEX, 3-MA, or RAPA-treated BMSCs. (**H-I**) Representative images of TUNEL staining and Annexin V-FITC/PI assay in 1 μM DEX, 3-MA, or RAPA-treated BMSCs. (**J**) ALP staining images of BMSCs treated with 1 μM DEX, 3-MA, or RAPA for 14 days. Data are presented as mean ± SD. NS no significance, *P < 0.05, **P < 0.01, and ***P < 0.001 (vs. DEX).

Given recent evidence that ferroptosis can be an autophagy-dependent process 38, 39 and that excessive autophagy exacerbates GC-induced apoptosis 40, 41, we hypothesized that the GC-induced suppression of osteogenesis in BMSCs is driven by autophagy-dependent ferroptosis and apoptosis. We utilized pharmacological tools to test this causal relationship directly. Inhibiting autophagy with 3-MA concurrently suppressed DEX-induced ferroptosis and apoptosis, while inducing autophagy with RAPA amplified both processes (Figure 4A-I, S3A-D), demonstrating that autophagy acts upstream. The pivotal finding was that this autophagy-dependent cell death was the direct cause of the impaired osteogenesis. The suppression of osteogenic differentiation by DEX was reversed by 3-MA and worsened by RAPA (Figure 4J, S3E). This functional rescue experiment provides direct evidence that autophagy is not merely coincidental. Nevertheless, it is the mechanistic linchpin because of which GC elevates ferroptosis and apoptosis, ultimately disrupting the osteogenic differentiation of BMSCs.

### NAR rescues GONFH phenotype by targeting autophagy-dependent ferroptosis, apoptosis, and osteogenic impairment

**Figure 5:**
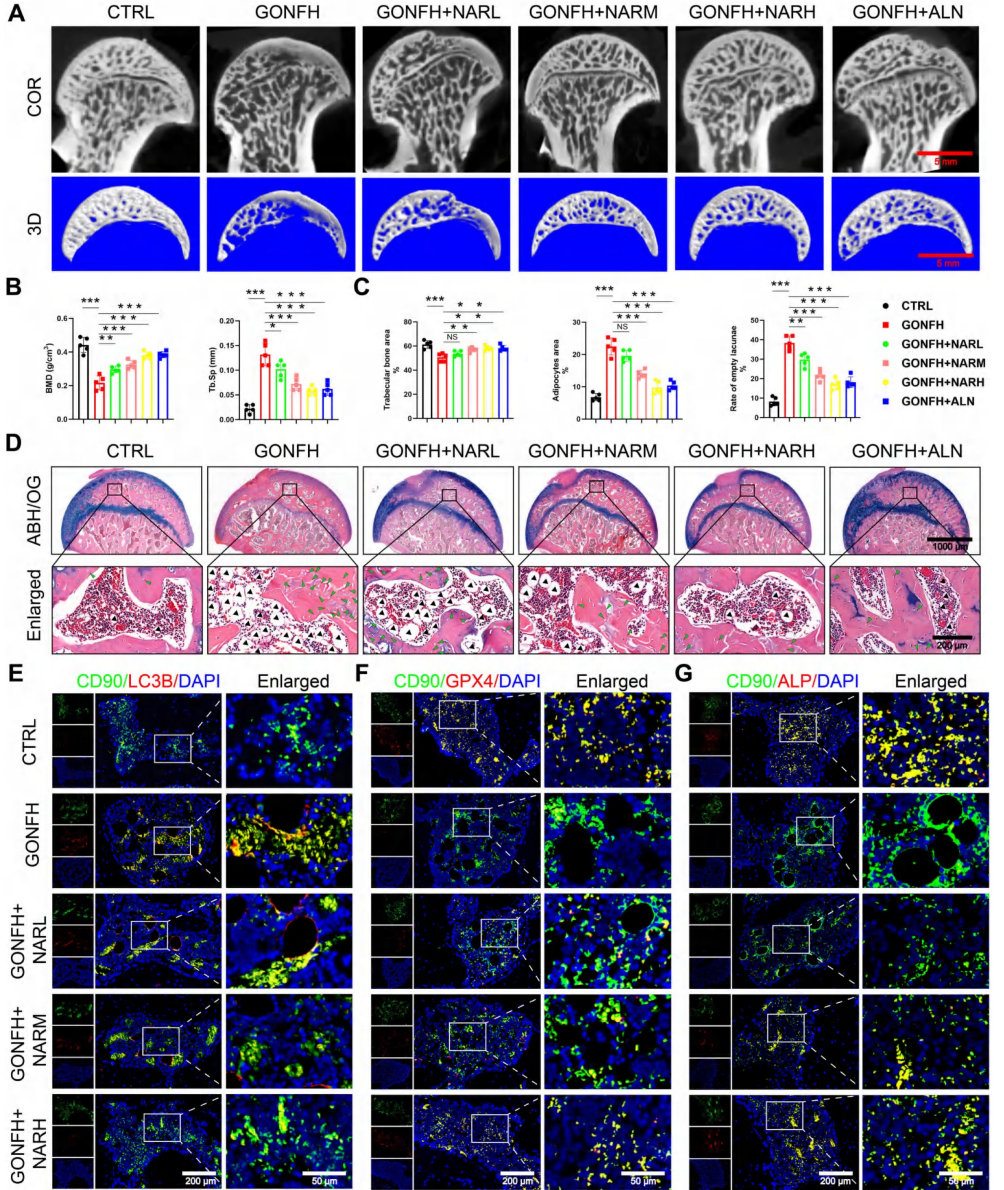
NAR notably represses autophagy-dependent ferroptosis and apoptosis while promoting the osteogenic differentiation of BMSCs to ameliorate the GONFH phenotype in rats. (**A**) Representative *μ*-CT scanning images of rat femoral head samples. (**B**) Quantitative analysis of BMD and Tb.Sp of rat femoral head samples (n = 5). (**C-D**) Representative histological images (ABH/OG) and histomorphometric analysis of rat femoral head samples (n = 5). The adipocytes are indicated by black arrows, and the empty lacunae are indicated by green arrows. (**E-G**) Representative IF co-staining images of CD90 and LC3B/GPX4/ALP of rat femoral head samples. Data are presented as mean ± SD. NS no significance, *P < 0.05, **P < 0.01, and ***P < 0.001 (vs. GONFH).

We then examined the therapeutic efficacy of NAR *in vivo*. Figure S4A demonstrates the experimental procedure with animals. NAR treatments in GONFH rats resulted in a dose-dependent increase in femoral head load-bearing capacity and bone microstructure, and reversed the osteonecrosis phenotype as observed by *μ*-CT and ABH/OG staining (Figure 5A-D, S4B-D). RNA-sequencing indicated that mechanical links were implicated in the AMPK signaling pathway (Figure S5A). We thus analyzed the underlying pathological pathway identified by us. NAR inhibited the activated autophagy in BMSCs, which was indicated by low LC3B/CD90 levels and high levels of P62 (Figure 5E, S5B). Furthermore, downstream ferroptosis and apoptosis were also alleviated (Figure 5F, 5C-D). Eventually, autophagy-dependent ferroptosis and apoptosis, which were inhibited by the situation, were rescued in BMSCs, allowing for osteogenic differentiation (Figure 5G). Our findings reveal that NAR exhibits inhibitory properties on ferroptosis and apoptosis in BMSCs, inducing osteogenic differentiation by regulating the AMPK-autophagy axis.

### NAR directly binds to ULK1 and promotes ser757 phosphorylation

**Figure 6:**
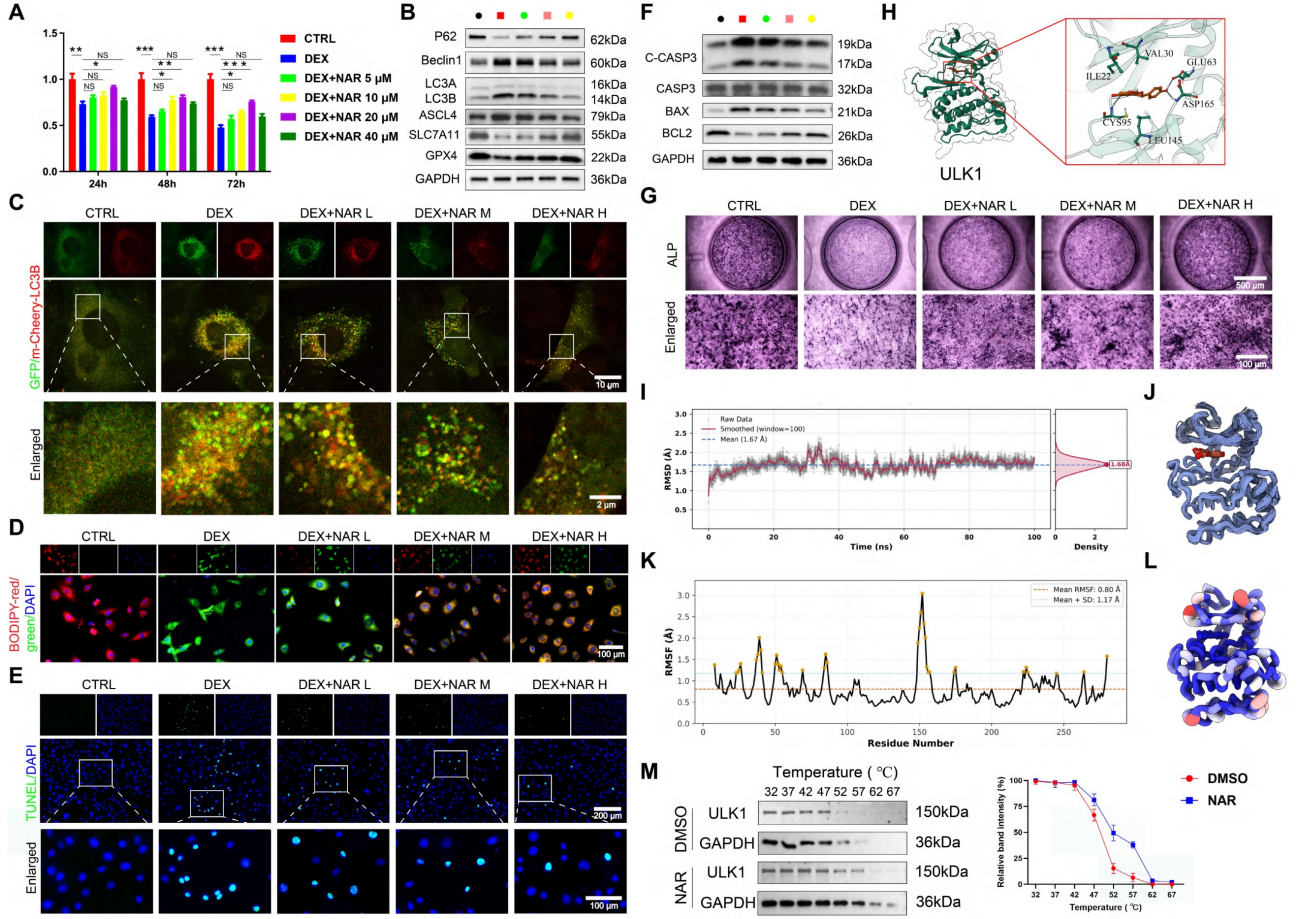
Increased phosphorylation of ULK1 ser757 by NAR ameliorates the reduction of autophagy-dependent ferroptosis and apoptosis-mediated osteogenic differentiation in BMSCs. (**A**) CCK8 assay for the proliferative capacity of BMSCs treated with 1 µM DEX and different concentrations of NAR (n = 3). (**B**) Protein levels of P62, Beclin1, LC3B, GPX4, SLC7A11 and ASCL4 in BMSCs treated with 1 µM DEX and different concentrations of NAR (n = 3). (**C**) Representative images of mCherry-GFP-LC3B in BMSCs treated with 1 µM DEX and different concentrations of NAR. (**D**) C11-BODIPY staining images in BMSCs treated with 1 µM DEX and different concentrations of NAR. (**E**) Representative images of TUNEL staining in BMSCs treated with 1 µM DEX and different concentrations of NAR. (**F**) Protein levels of C-CASP3, CASP3, BAX and BCL2 in BMSCs treated with 1 µM DEX and different concentrations of NAR (n = 3). (**G**) ALP staining images of BMSCs treated with 1 µM DEX and different concentrations of NAR for 14 days. (**H**) Molecular docking of NAR and ULK1. (**I**) RMSD analysis of ULK1-NAR complex. (**J**) ULK1-NAR trajectory overlay at the last 10ns of simulation. (**K**) RMSF analysis of ULK1-NAR complexes. (**L**) Factor diagram of ULK1-NAR complex. (**M**) CETSA analysis of the binding between ULK1 and NAR in BMSCs (n = 3). Data are presented as mean ± SD. NS no significance, *P < 0.05, **P < 0.01, and ***P < 0.001 (vs. DEX).

To further explain its working mechanism, we conducted follow-up experiments. CCK-8 analysis revealed that NAR reversed the effect of DEX on the proliferation of BMSCs in a dose-dependent manner at concentrations below 20 μM (Figure 6A). We, therefore, chose low, medium, and high dose groups of 5, 10, and 20 μM as dose groups to be utilized in future studies. We found that NAR dose-dependently suppressed DEX-induced autophagy, which in turn inhibited subsequent ferroptosis and apoptosis, and restored osteogenic differentiation (Figure 6B-G, S6).

Additionally, molecular docking revealed that NAR (binding energy: -9.03 kcal/mol) is a higher-affinity target of ULK1 than AMPK or MTOR (Figure 6H, S7A-B). The specificity and stability of NAR-ULK1 interaction were stabilized and confirmed by molecular dynamics simulations and CETSA (Figure 6I-M, S7C-G). Moreover, WB analysis revealed that NAR's action is mediated by phosphorylation, as it upregulates inhibitory phosphorylation of ULK1 at ser757 without altering the phosphorylation status of AMPK, MTOR, or the ULK1 activation site (ser555) (Figure S7H). Our data demonstrate that NAR directly engages ULK1 and acts as a molecular switch to promote its inhibitory phosphorylation at ser757, which in turn curtails the pathological autophagy-dependent ferroptosis and apoptosis responsible for impaired osteogenesis.

### Inhibition of ULK1 ser757 phosphorylation abrogates the therapeutic effects of NAR in BMSCs

**Figure 7:**
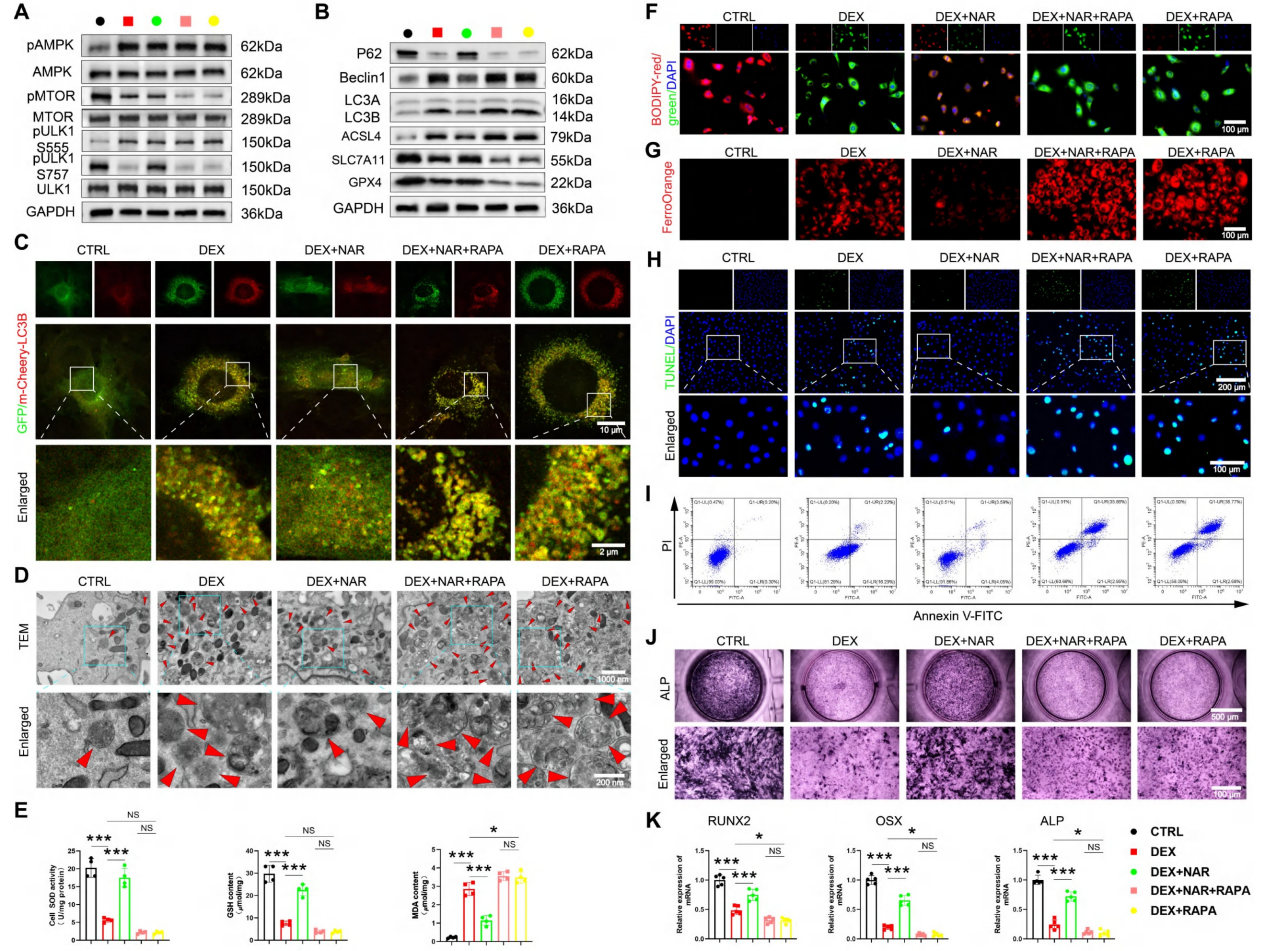
RAPA abrogates the effects of NAR on autophagy-dependent ferroptosis, apoptosis, and osteogenic differentiation in BMSCs. (**A**) The levels of AMPK/MTOR/ULK1 signaling pathway-related proteins in BMSCs treated with 1 µM DEX, 20 µM NAR and RAPA (n = 3). (**B**) Protein levels of P62, Beclin1, LC3B, GPX4, SLC7A11 and ASCL4 in BMSCs treated with 1 µM DEX, 20 µM NAR and RAPA (n = 3). (**C**) Representative images of mCherry-GFP-LC3B in BMSCs treated with 1 µM DEX, 20 µM NAR and RAPA. (**D**) Representative TEM images of BMSCs treated with 1 µM DEX, 20 µM NAR and RAPA. (**E**) SOD, GSH and MDA levels in BMSCs treated with 1 µM DEX, 20 µM NAR and RAPA (n = 4). (**F-G**) C11-BODIPY and FerroOrange staining images in BMSCs treated with 1 µM DEX, 20 µM NAR and RAPA. (**H-I**) Representative images of TUNEL staining and Annexin V-FITC/PI assay in BMSCs treated with 1 µM DEX, 20 µM NAR and RAPA. (**J**) ALP staining images of BMSCs treated with 1 µM DEX, 20 µM NAR and RAPA for 14 days. (**K**) mRNA levels of RUNX2, OSX and ALP in BMSCs treated with 1 µM DEX, 20 µM NAR and RAPA (n = 5). Data are presented as mean ± SD. NS no significance, *P < 0.05, and ***P < 0.001 (vs. DEX or DEX+RAPA).

We tested the requirement of ULK1 ser757 phosphorylation in NAR's action through two complementary approaches. RAPA inhibits MTOR activity, thereby activating autophagy through the dephosphorylation of the MTOR site and the inhibition of the suppressive phosphorylation site ser757 of ULK1 42. First, using RAPA to pharmacologically inhibit this specific phosphorylation, we found that RAPA had no notable effect on pAMPK and pULK1 ser555 levels (Figure 7A, Figure S8A). However, when RAPA was coupled with NAR, pMTOR and pULK1 ser757 levels were remarkably diminished compared to the DEX+NAR group and were comparable to those in the DEX+RAPA group (Figure 7A, S8A). In addition, RAPA completely reversed NAR's suppression of autophagy-dependent ferroptosis and apoptosis, as well as its restoration of osteogenesis (Figure 7B-K, S8B-F).

**Figure 8:**
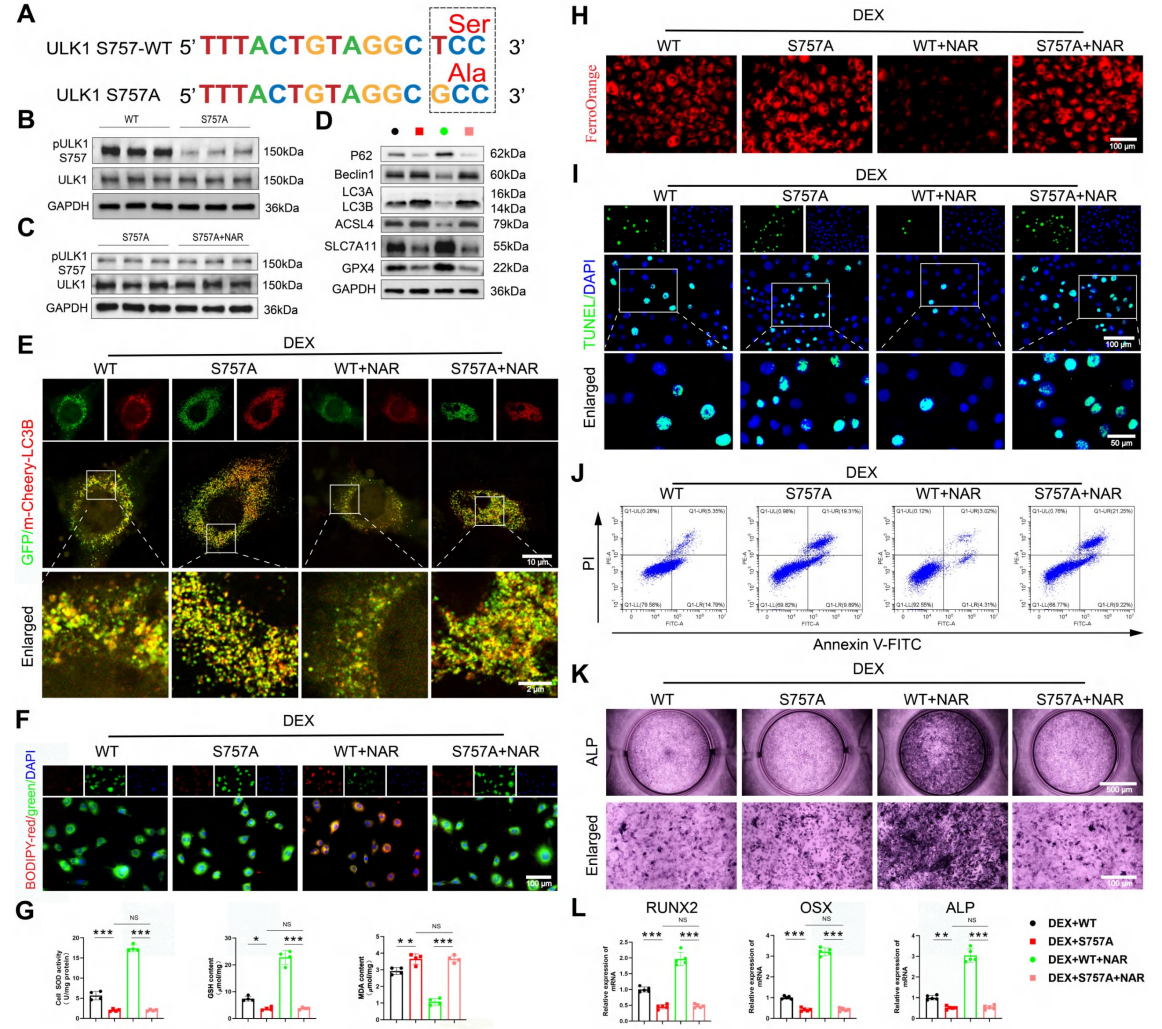
The mutation of ULK1 ser757 eliminates the effects of NAR on autophagy-dependent ferroptosis, apoptosis, and osteogenic differentiation in BMSCs. (**A**) Mutation site for ULK1 ser757. (**B-C**) Protein levels of pULK1 ser757 and ULK1 in BMSCs treated with S757A and NAR (n = 3). (**D**) Protein levels of P62, Beclin1, LC3B, GPX4, SLC7A11 and ASCL4 in BMSCs treated with 1 µM DEX and WT or S757A or 20 µM NAR (n = 3). (**E**) Representative images of mCherry-GFP-LC3B in BMSCs treated with 1 µM DEX and WT or S757A or 20 µM NAR. (**F, H**) C11-BODIPY and FerroOrange staining images in BMSCs treated with 1 µM DEX and WT or S757A or 20 µM NAR. (**G**) MDA, GSH and SOD levels in BMSCs treated with 1 µM DEX and WT or S757A or 20 µM NAR (n = 4). (**I-J**) Representative images of TUNEL staining and Annexin V-FITC/PI assay in BMSCs treated with 1 µM DEX and WT or S757A or 20 µM NAR. (**K**) ALP staining images of BMSCs treated with 1 µM DEX and WT or S757A or 20 µM NAR for 14 days. (**L**) mRNA levels of RUNX2, OSX and ALP in BMSCs treated with 1 µM DEX and WT or S757A or 20 µM NAR (n = 5). Data are presented as mean ± SD. NS no significance, *P < 0.05, **P < 0.01, and ***P < 0.001 (vs. WT or DEX+S757A or DEX+S757A+NAR).

Second, to rule out off-target effects and provide definitive proof, we constructed an overexpression virus with the ULK1 ser757 mutation, and the specific mutation site is shown in Figure 8A. We validated the mutation efficiency and found that the pULK1 ser757 was notably decreased in the S757A group compared to the wild-type (WT) group (Figure 8B, Figure S9A). In addition, we found that mutation of ULK1 ser757 eliminated the promotion of pULK1 ser757 by NAR (Figure 8C, Figure S9B). Notably, the genetic mutation of this phosphorylation site completely abolished the NAR's downstream benefits, as autophagy remained elevated, ferroptosis and apoptosis were unchecked, and osteogenic differentiation failed to recover (Figure 8D-L). Together, the convergence of pharmacological and genetic evidence establishes that ULK1 ser757 phosphorylation is not merely associated with, but is strictly required for, NAR's therapeutic efficacy in rescuing the osteogenic differentiation of BMSCs.

### RAPA abolishes the therapeutic efficacy of NAR in GONFH rats

**Figure 9:**
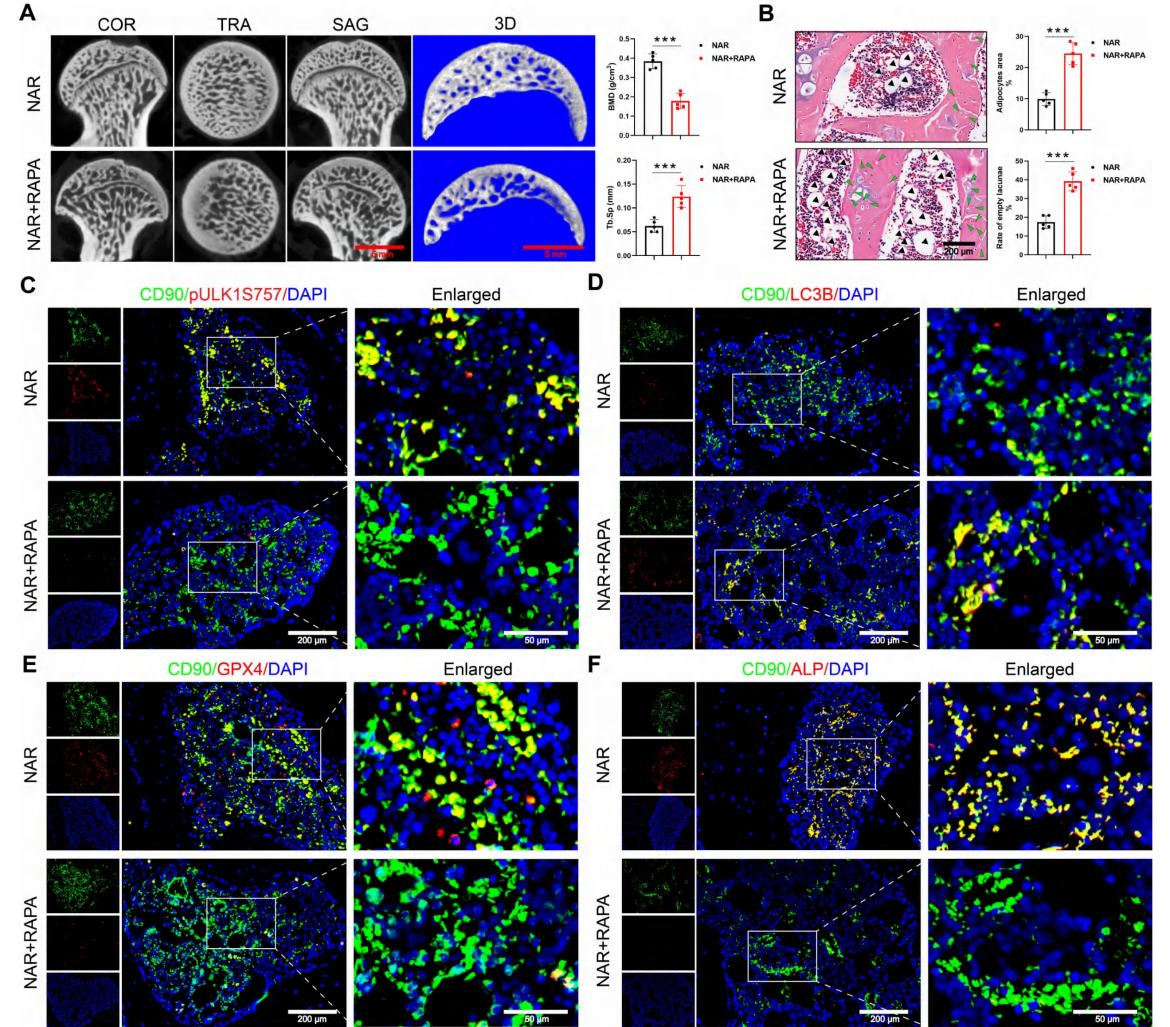
RAPA abolishes the therapeutic effect of NAR in GONFH rats. (**A**) Representative *μ*-CT scanning images and quantitative analysis of BMD and Tb.Sp of rat femoral head samples (n = 5). (**B**) Representative histological images (ABH/OG) and histomorphometric analysis of rat femoral head samples (n = 5). The adipocytes are indicated by black arrows, and the empty lacunae are indicated by green arrows. (**C-F**) Representative IF co-staining images of CD90 and pULK1 ser757/LC3B/GPX4/ALP of rat femoral head samples. Data are presented as mean ± SD. ***P < 0.001 (vs. NAR).

We conducted a pivotal in vivo experiment to determine whether NAR's therapeutic effects are mediated through the RAPA-sensitive ULK1 ser757 phosphorylation pathway. The specific animal experimental procedures are illustrated in Figure S10A. In GONFH rats, the co-administration of RAPA completely abolished the therapeutic benefits of NAR. Specifically, RAPA reversed NAR-mediated improvements in femoral head load-bearing capacity, bone microarchitecture, and histopathology (Figure 9A, B, S10B-D).

At the molecular level, RAPA co-treatment prevented the NAR-induced upregulation of pULK1 (ser757) and subsequently reversed NAR's inhibitory effects on autophagy-dependent ferroptosis and apoptosis (Figure 9C-E, S10E, F). As a final consequence, the restoration of BMSCs' osteogenic differentiation by NAR was also negated in the presence of RAPA (Figure 9F). This set of in vivo evidence conclusively shows that NAR's capacity to treat GONFH relies entirely on a signaling axis that is disrupted by RAPA, providing strong functional support for the proposed mechanism involving ULK1 ser757.

## Discussion

This research paper aimed to clarify one of the novel pathogenic mechanisms of GONFH and to explore the therapeutic possibilities of NAR. We ensured that excessive GC stimulates AMPK/MTOR/ULK1 signaling pathway, and hence excessive autophagy in BMSCs. Interestingly, we found that overindulgence of autophagy in BMSCs promotes ferroptosis and apoptosis, ultimately leading to decreased osteogenic differentiation and deterioration of GONFH. We also noted that NAR is an exciting putative therapeutic that can directly interfere with ULK1, thereby inducing inhibitory phosphorylation of ser757 to block this pathogenic cascade as a treatment of GONFH.

The disrupted osteogenic differentiation of BMSCs is widely accepted as a major cause of GONFH pathogenesis 3, 4. Autophagy, which is a two-edged sword, is not well comprehended with respect to GONFH. Past literature suggests that GONFH is associated with insufficient autophagy, and stimulating autophagy is recommended for its treatment 43, 44. However, contrary to this, our results indicate that excessive autophagy of BMSCs, mediated by GC, is a risk factor for GONFH. In a physiological state, autophagy is a vital cellular defense process required to sustain the homeostasis and osteogenic ability of BMSCs. Autophagy deficiencies lead to cellular dysfunction. On the other hand, in case of extreme or prolonged pathological damage, e.g., intense GC exposure, autophagy is overactivated and insane 45-47. Therefore, opposite results may be the outcome of different levels of experimentation. Since researchers on autophagy-promoting treatments for illness tend to utilize autophagy-deficient models, our study uses high-concentration GC to induce autophagy to an overactivated phase. This difference is critical in targeted therapies, implying that the treatment of a disease should specifically repair the systems of autophagy, which is suitable for the disease progression and cell landscape. Thus, the application of RAPA to GONFH was found to exacerbate it in our study, although RAPA had previously been shown to have positive outcomes for relevant orthopedic conditions 48-50. The root cause of this radically different effect may be that, in cases such as osteoarthritis or bone loss, autophagy is deficient, and accordingly, may lead to cellular senescence or degradation of the matrix. Conversely, elevated autophagy has therapeutic implications. Conversely, in our GONFH model, autophagy is already hyperactivated, and it is not surprising that RAPA exacerbates GONFH. This demonstrates that the therapeutic potential of autophagy modulators is not absolute but depends on the specific pathological context and preexisting levels of autophagy activity.

Our analysis has shown that excessive autophagy is a mediating factor that triggers GC-induced ferroptosis and apoptosis in BMSCs. This is consistent with recent evidence indicating that iron homeostasis and lipid peroxidation, facilitated by autophagy, promote ferroptosis 39, 51, 52 and facilitate apoptosis by degrading anti-apoptotic proteins 14, 53. The use of autophagy modulators 3-MA and RAPA also proved that the high levels of ferroptosis and apoptosis in BMSCs are the downstream consequences of autophagy stimulation, thereby refining the pathogenic model of GONFH.

AMPK is a key regulator of autophagy 54, 55. In nutrient-sufficient settings, the inhibition of AMPK triggers the activation of MTOR, which in turn inhibits ULK1 phosphorylation (e.g., ser757), resulting in the inability of ULK1 to form the initiation complex and thus inhibiting autophagy 55, 56. Conversely, under energy stress, AMPK activation inhibits MTOR, leading to ULK1 phosphorylation (e.g., at ser555) and thereby initiating autophagy 57. Our results confirm the importance of this signaling pathway to GONFH, and we have found that the AMPK/MTOR/ULK1 signaling cascade, known to be excessively triggered by GC, promotes a shift in the balance towards increased autophagy.

At present, GONFH does not have a general medicine. Despite demonstrating therapeutic effects as exhibited by bisphosphonates (BPs, including ALN), their possible side effects, such as renal impairment, cannot be avoided 58-60. NAR, a natural flavonoid known for its anti-inflammatory and bone-protective properties 21, 22, 25, 61, 62, represents a potential therapeutic agent. We found that NAR has therapeutic effects via binding to ULK1, as confirmed by molecular docking, dynamics simulation, and CETSA. NAR promotes the anti-autophagic inhibitory phosphorylation of ULK1 at ser757, which inhibits excessive autophagy in BMSCs and induces ferroptosis and apoptosis, ultimately facilitating osteogenic differentiation. It is worth noting that NAR has been shown to exhibit better therapeutic activity in vivo compared to ALN, particularly at a dose of 20 mg/kg/day, which underscores its clinical advantages in arresting or prolonging GONFH.

Studies have established that GC influences bone angiogenesis and metabolic balance, which are important factors in the pathogenesis of GONFH 63, 64. High-dose GC inhibits angiogenesis by suppressing vascular endothelial growth factor (VEGF) and promoting endothelial cell dysfunction, thereby impairing blood supply to the femoral head and accelerating disease progression 63, 65. Furthermore, excessive GC shifts the differentiation of BMSCs from osteogenesis toward adipogenesis, leading to reduced trabecular bone and diminished bone mass in necrotic areas 4, 66. The present work demonstrates that GC in high dosage triggers autophagy-dependent ferroptosis and apoptosis, while also inhibiting osteogenesis. This finding aligns with and potentially contributes to vascular degradation and bone metabolic dysfunction. An example is that excessive autophagy activation can lead to ferroptosis and apoptosis in endothelial or osteoblast cells under high glucose stress, potentially compromising vascular integrity or matrix production. Subsequent research must be systematic and employ research mechanisms to determine the modulatory effects of various GC dosages on autophagy, cell death, angiogenesis, and bone metabolism, thereby gaining a deeper understanding of the pathogenesis of GONFH and supporting the development of a therapeutic strategy.

Despite this study demonstrating that NAR can salvage the role of BMSCs and ameliorate GONFH, several important issues related to translation need to be addressed. Due to its poor solubility and high rate of metabolism, NAR exhibits poor oral bioavailability, making nanocrystals or lipid carriers viable alternatives. Second, the route of administration matters (e.g., local delivery, such as intraosseous injection or integration into biomaterial reservoirs) improves local delivery to the femoral head. Incorporating NAR into injectable hydrogels or bone scaffolds enables sustained release while providing bone-inductive support. The next direction of future research should be the application of NAR in combination with osteoinductive agents or cell therapies to have a synergistic effect on bone repair.

The limitations of our study are as follows. First, we have primary clinical evidence on patients with advanced GONFH, but the animal model replicates early-stage GONFH. Despite the usefulness of the LPS+MPS rodent model in studying acute inflammation and metabolic dysregulation, a chronic course and progressive structural failure, as well as reparative responses, are not achieved in the rodent model compared to the situation in human GONFH. The closest animal model of the disease replicates the whole spectrum of the disease. Consequently, to evaluate the role of autophagy, which may be active throughout the disease or only in certain stages, we need to prepare an animal model that completely recreates the entire GONFH disease pathway, thus clarifying the active role of autophagy in all disease phases. Such methods can be: (1) large animal models to strengthen structural and long-term studies; (2) Models that trigger therapeutic interventions following exposure to GC to simulate clinically secondary osteonecrosis; (3) Longitudinal analysis to track the progression of early necrosis to collapse.

## Conclusions

**Figure 10:**
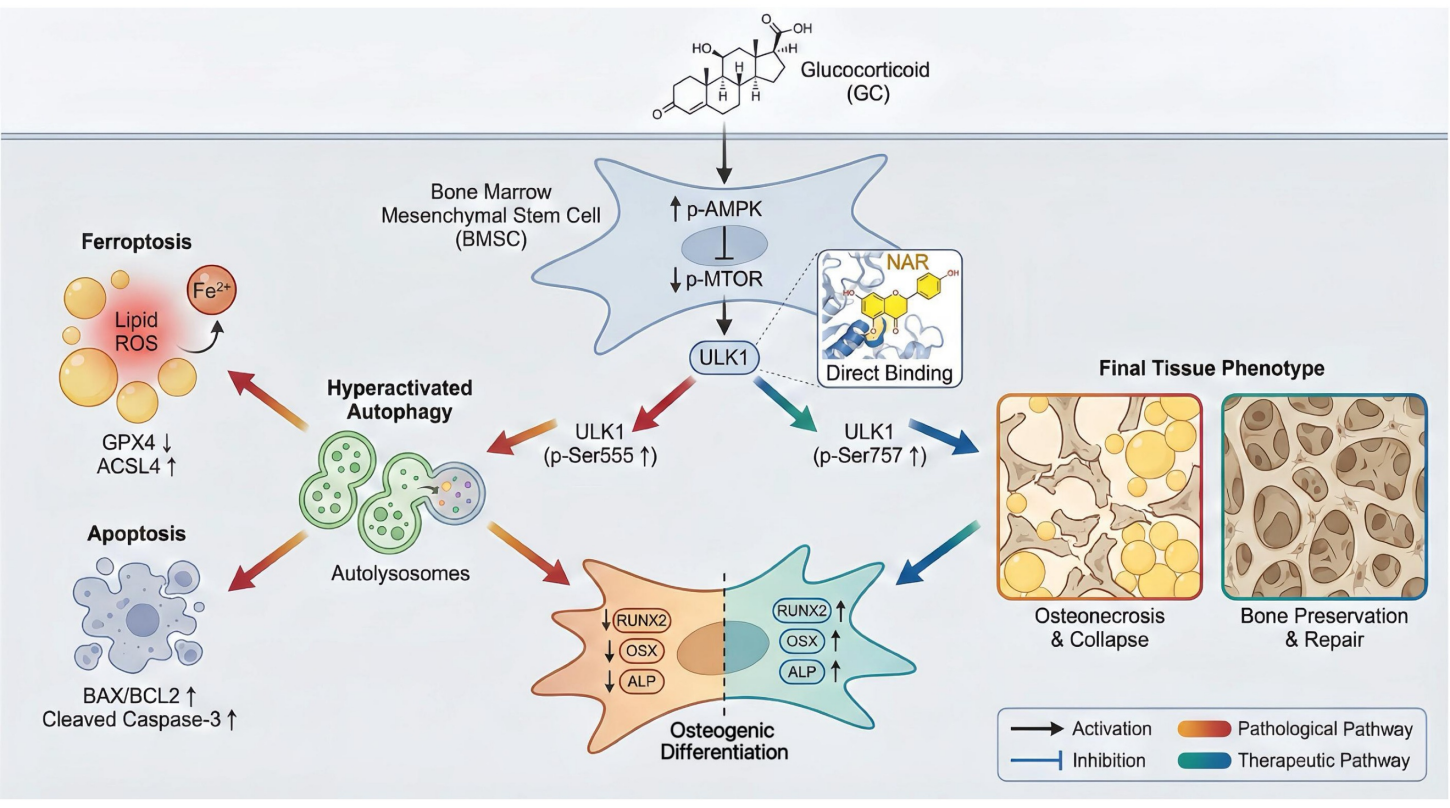
Schematic illustration of the underlying protective role of NAR in the development of GONFH. GC-impaired osteogenesis in BMSCs is mediated by autophagy-dependent ferroptosis and apoptosis via the AMPK/MTOR/ULK1 pathway, revealing that NAR alleviates GONFH by enhancing ULK1 phosphorylation at ser757 to inhibit this detrimental cascade.

To conclude, the present article shows that high concentrations of GC stimulate the AMPK/MTOR/ULK1 signaling pathway, worsen autophagy-dependent ferroptosis and apoptosis, and impair the osteogenic differentiation of BMSCs. We also consider NAR a potent therapeutic agent for repressing ferroptosis and apoptosis, which are dependent on autophagy in GONFH, by inhibiting ULK1 and facilitating ser757 phosphorylation of ULK1 (Figure 10).

## Supplementary Material

Supplementary figures and tables.

## Data Availability

The datasets used and/or analyzed during the current study are available from the corresponding author on reasonable request.

## Abbreviations

3-MA: 3-methyladenine
ABH/OG: alcian blue haematoxylin/orange g
ALN: alendronate
ALP: alkaline phosphatase
BAX: B-cell lymphoma 2 (BCL2)-associated X protein
BCL2: B-cell lymphoma 2
BMD: bone mineral density
BMSCs: bone marrow mesenchymal stem cells
BV/TV: trabecular bone volume fraction
CASP3: caspase-3
C-CASP3: cleaved caspase-3
CCK-8: cell counting kit-8
CETSA: cellular thermal shift assay
DEX: dexamethasone
CTRL: control
DAPI: 4',6-diamidino-2-phenylindole
FNF: femoral neck fracture
GAPDH: glyceraldehyde-3-phosphate dehydrogenase
GC: glucocorticoid
GONFH: glucocorticoid-associated osteonecrosis of the femoral head
GPX4: glutathione peroxidase 4
GSH: glutathione
IF: immunofluorescence
IHC: immunohistochemistry
KEGG: kyoto encyclopedia of genes and genomes
LPS: lipopolysaccharide
MDA: malondialdehyde
MPS: methylprednisolone
NAR: naringenin
ONFH: osteonecrosis of the femoral head
OSX: osterix
RAPA: rapamycin
RCD: regulated cell death
ROS: reactive oxygen species
RT-qPCR: reverse transcription-quantitative PCR
RUNX2: runt-related transcription factor 2
SOD: superoxide dismutase
Tb.Sp: trabecular bone separation
Tb.Th: trabecular thickness
Tb.N: trabecular bone number
TEM: transmission electron microscopy
TUNEL: Terminal-deoxynucleoitidyl Transferase Mediated Nick End Labeling
WB: western blotting
