# Supplementary material for: From pathogenesis to treatment: the role of autophagic cell death in GONFH and its potential mitigation by naringenin
Source: Theranostics. 2026 Feb 18;16(9):4804–20. doi: 10.7150/thno.129809 (PMC12964242; doi:10.7150/thno.129809)
Supplement: Supplementary file 1 — Supplementary figures and tables. [file thnov16p4804s1.pdf]

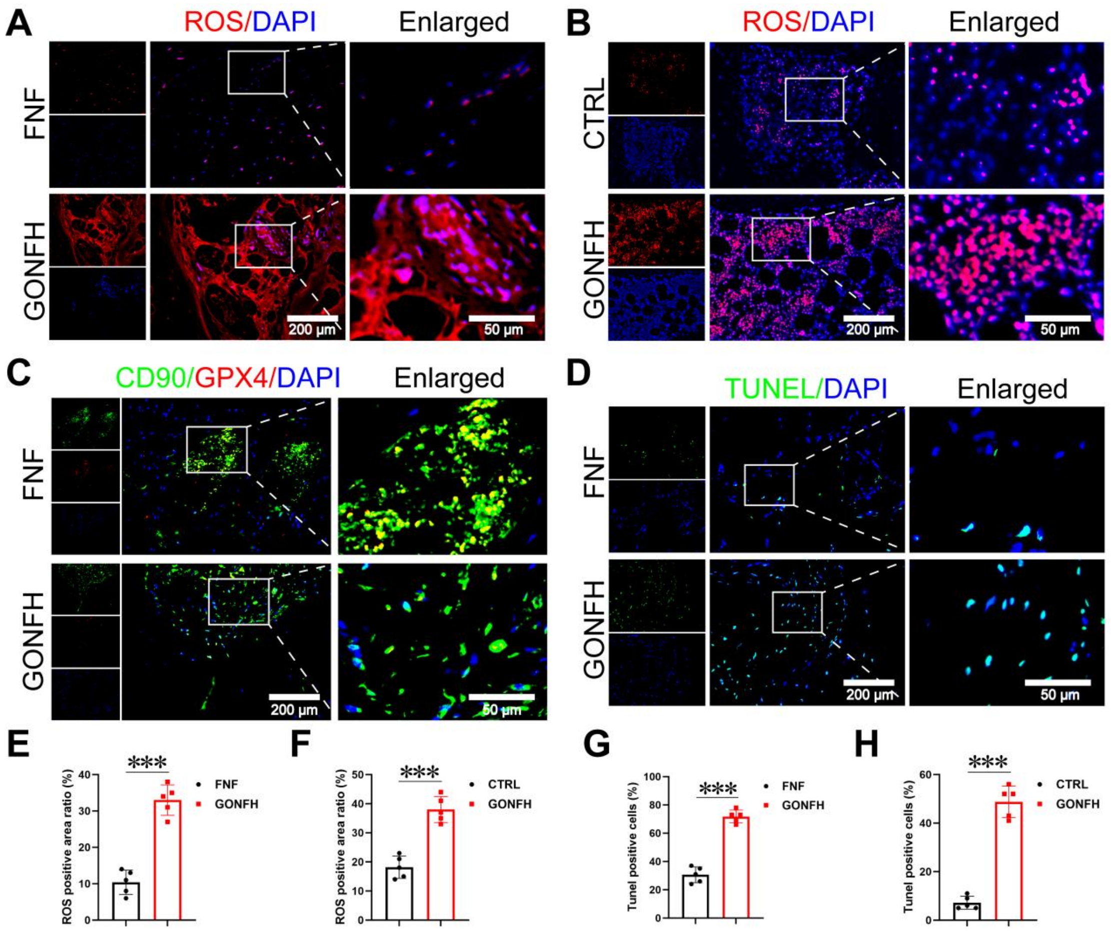

Figure S1. (**A-B**) Representative ROS staining images of human and rat femoral head samples. (**C**) Representative IF co-staining images of CD90 and GPX4 of human femoral head samples. (**D**) Representative TUNEL staining images of human femoral head samples. (**E-F**) Quantitative analysis of ROS staining of human and rat femoral head samples (n = 5). (**G-H**) Quantitative analysis of TUNEL staining of human and rat femoral head samples (n = 5). Data are presented as mean ± SD. ****P* < 0.001 (vs. GONFH).

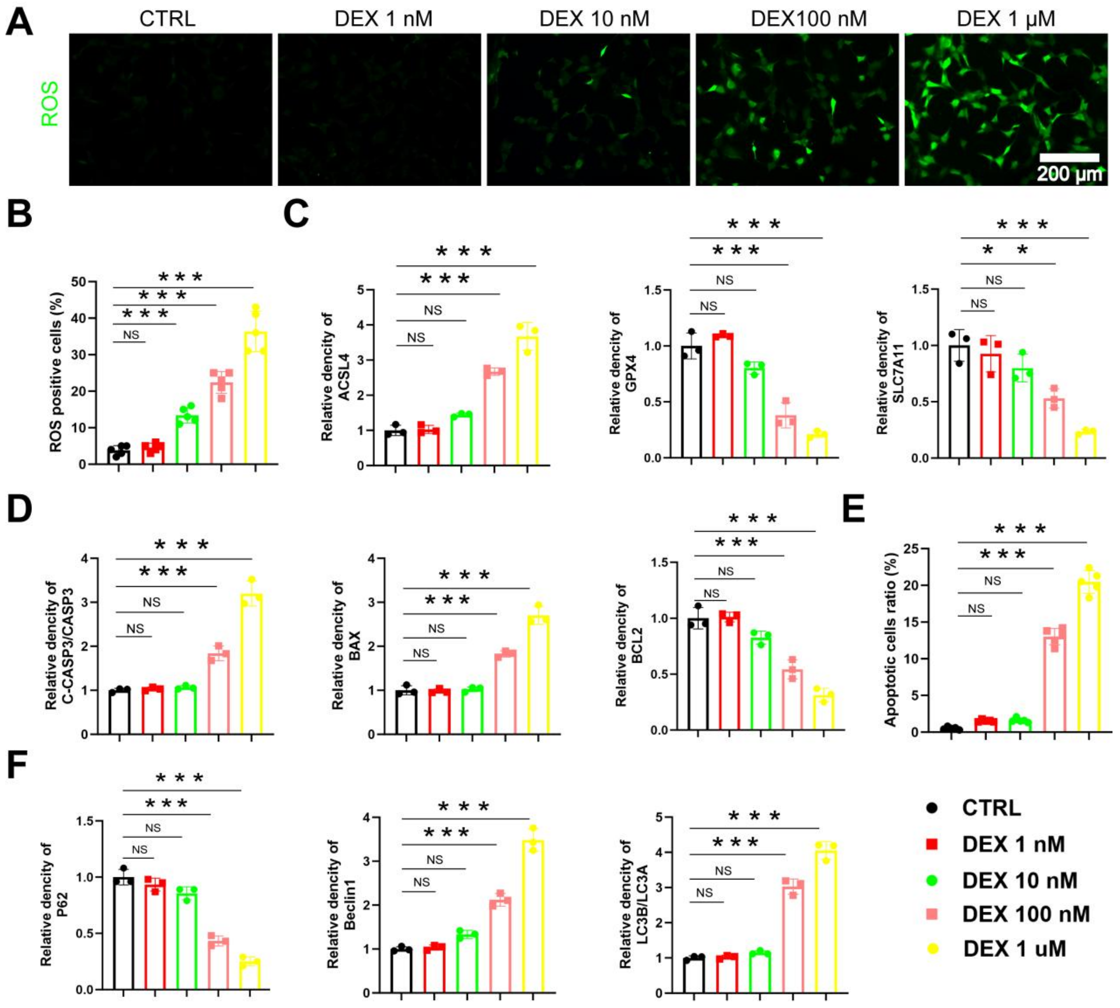

Figure S2. (**A-B**) Representative ROS staining images and quantitative analysis in BMSCs treated with different concentrations of DEX (n = 5). (**C**) Protein levels of ASCL4, GPX4 and SLC7A11 in BMSCs treated with different concentrations of DEX (n = 3). (**D**) Protein levels of C-CASP3, CASP3, BAX and BCL2 in BMSCs treated with different concentrations of DEX (n = 3). (**E**) Apoptotic ratio of BMSCs treated with different concentrations of DEX (n = 5). (**F**) Protein levels of P62, Beclin1 and LC3B in BMSCs treated with different concentrations of DEX (n = 3). Data are presented as mean ± SD. NS no significance, $**P < 0.01$, and $***P < 0.001$ (vs. CTRL).

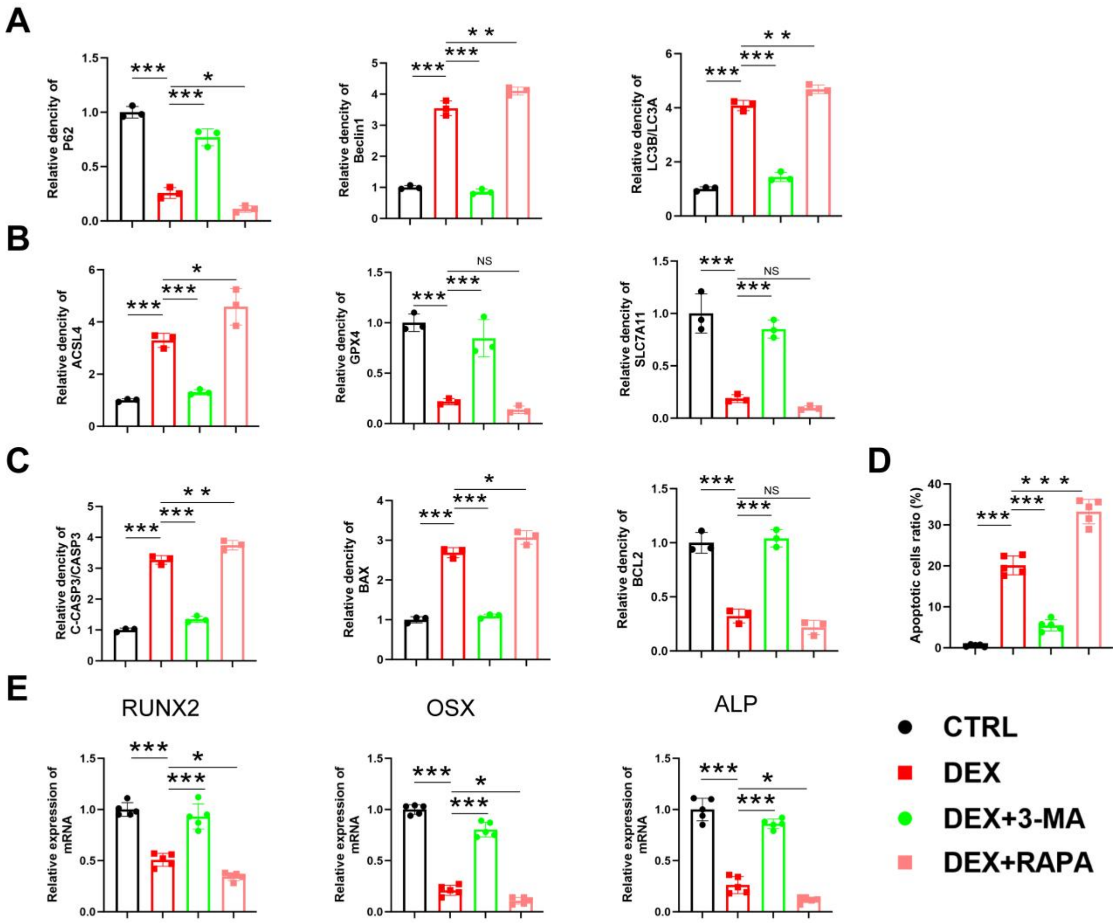

Figure S3. (**A-C**) Protein levels of P62, Beclin1, LC3B, ASCL4, GPX4, SLC7A11, C-CASP3, CASP3, BAX and BCL2 in 1 μM DEX or 3-MA or RAPA-treated BMSCs (n = 3). (**D**) Apoptotic ratio in 1 μM DEX or 3-MA or RAPA-treated BMSCs (n = 5). (**E**) mRNA levels of RUNX2, OSX and ALP in 1 μM DEX or 3-MA or RAPA-treated BMSCs (n = 5). Data are presented as mean ± SD. NS no significance, *$P < 0.05$, **$P < 0.01$, and ***$P < 0.001$ (vs. 1 μM DEX).

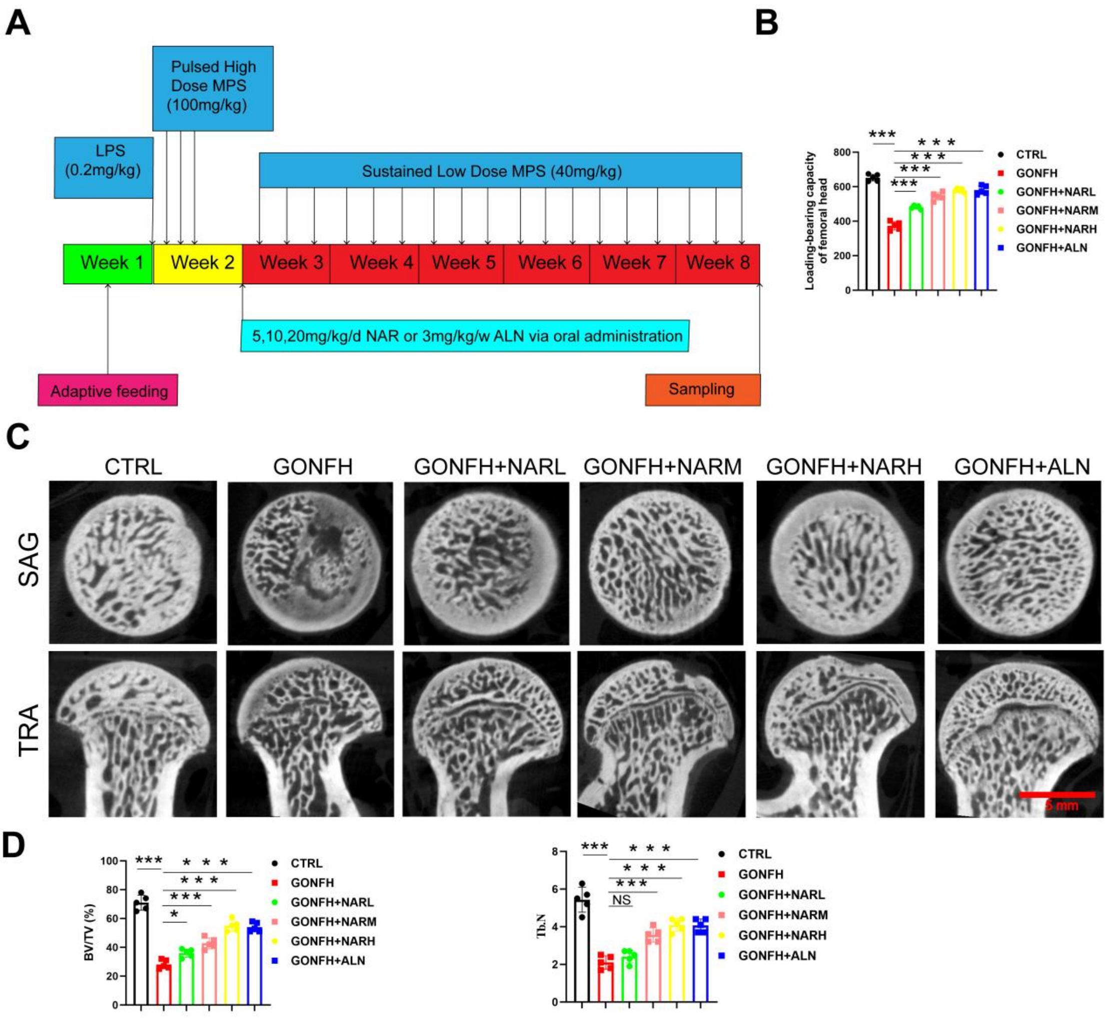

Figure S4. (**A**) The schematic diagram of the in vivo modelling procedure. (**B**) The load-bearing capacity of rat femoral head samples (n = 5). (**C**) Representative μ-CT scanning images of rat femoral head samples. (**D**) Quantitative analysis of BV/TV and Tb.N of rat femoral head samples (n = 5). Data are presented as mean ± SD. NS no significance, *P < 0.05, and ***P < 0.001 (vs. GONFH).

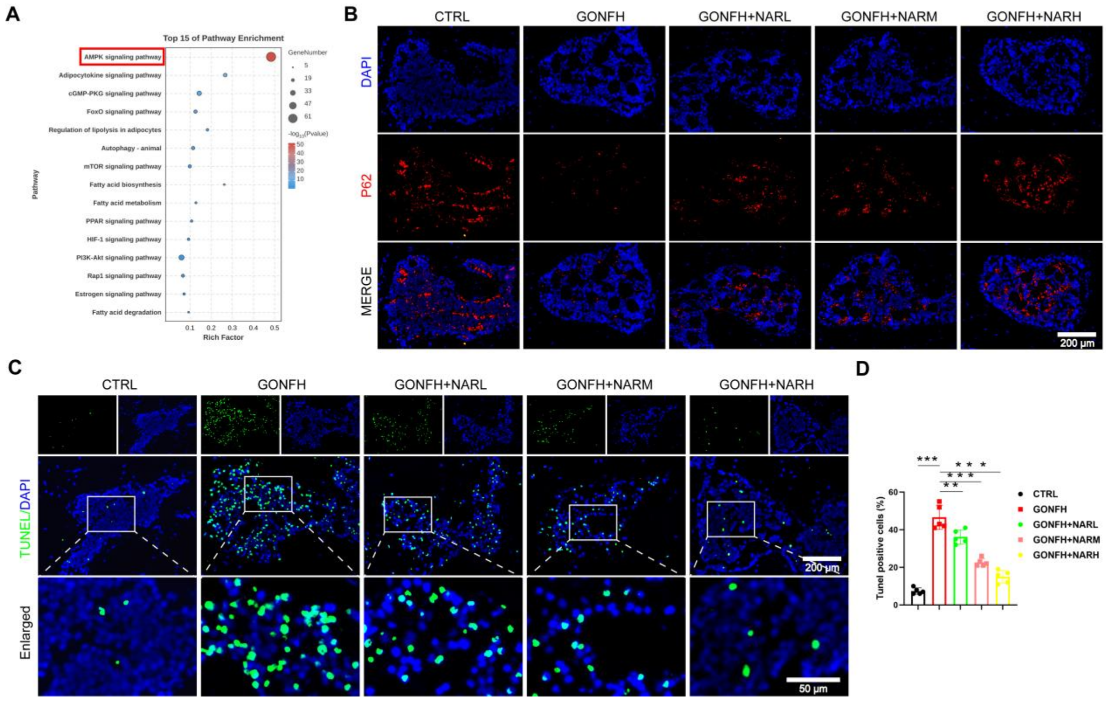

Figure S5. (**A**) KEGG enrichment analysis plot for RNA sequencing. (**B**) Representative IF staining images of P62 of rat femoral head samples. (**C-D**) Representative TUNEL staining images and quantitative analysis of rat femoral head samples (n = 5). Data are presented as mean ± SD. ***P* < 0.01, and ****P* < 0.001 (vs. GONFH).

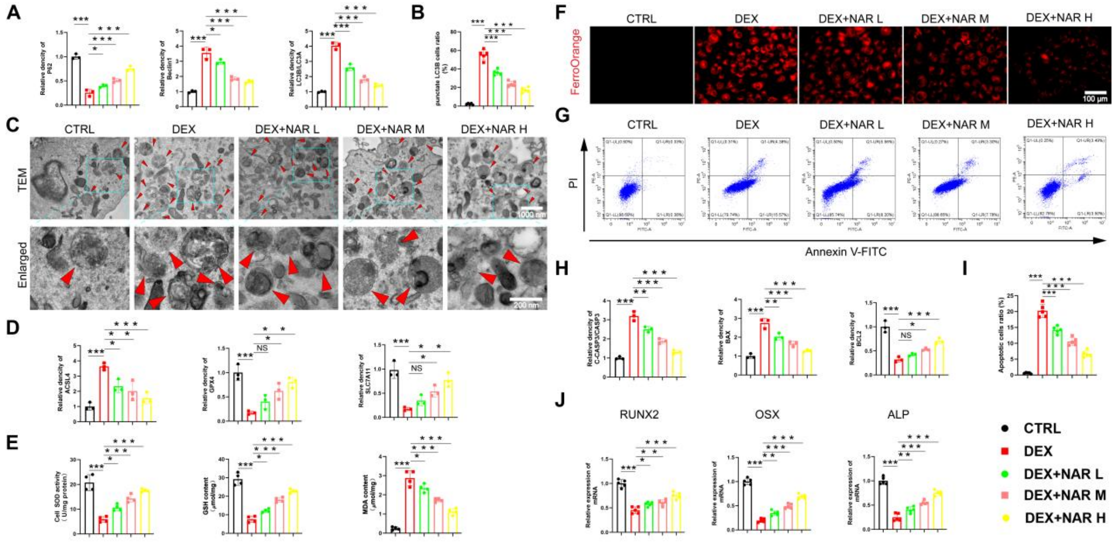

Figure S6. (**A**) Protein levels of P62, Beclin1 and LC3B in BMSCs treated with 1 μM DEX and different concentrations of NAR (n = 3). (**B**) Quantitative analysis of mCherry-GFP-LC3B in BMSCs treated with 1 μM DEX and different concentrations of NAR (n = 5). (**C**) Representative TEM images in BMSCs treated with 1 μM DEX and different concentrations of NAR. (**D**) Protein levels of ASCL4, GPX4 and SLC7A11 in BMSCs treated with 1 μM DEX and different concentrations of NAR (n = 3). (**E**) SOD, GSH and MDA levels in BMSCs treated with 1 μM DEX and different concentrations of NAR (n = 4). (**F**) FerroOrange staining images in BMSCs treated with 1 μM DEX and different concentrations of NAR. (**G**) Annexin V-FITC/PI assay in BMSCs treated with 1 μM DEX and different concentrations of NAR. (**H**) Protein levels of C-CASP3, CASP3, BAX and BCL2 in BMSCs treated with 1 μM DEX and different concentrations of NAR (n = 3). (**I**) Apoptotic ratio in BMSCs treated with 1 μM DEX and different concentrations of NAR (n = 5). (**J**) mRNA levels of RUNX2, OSX and ALP in BMSCs treated with 1 μM DEX and different concentrations of NAR (n = 5). Data are presented as mean ± SD. NS no significance, *$P < 0.05$, **$P < 0.01$, and ***$P < 0.001$ (vs. 1 μM DEX).

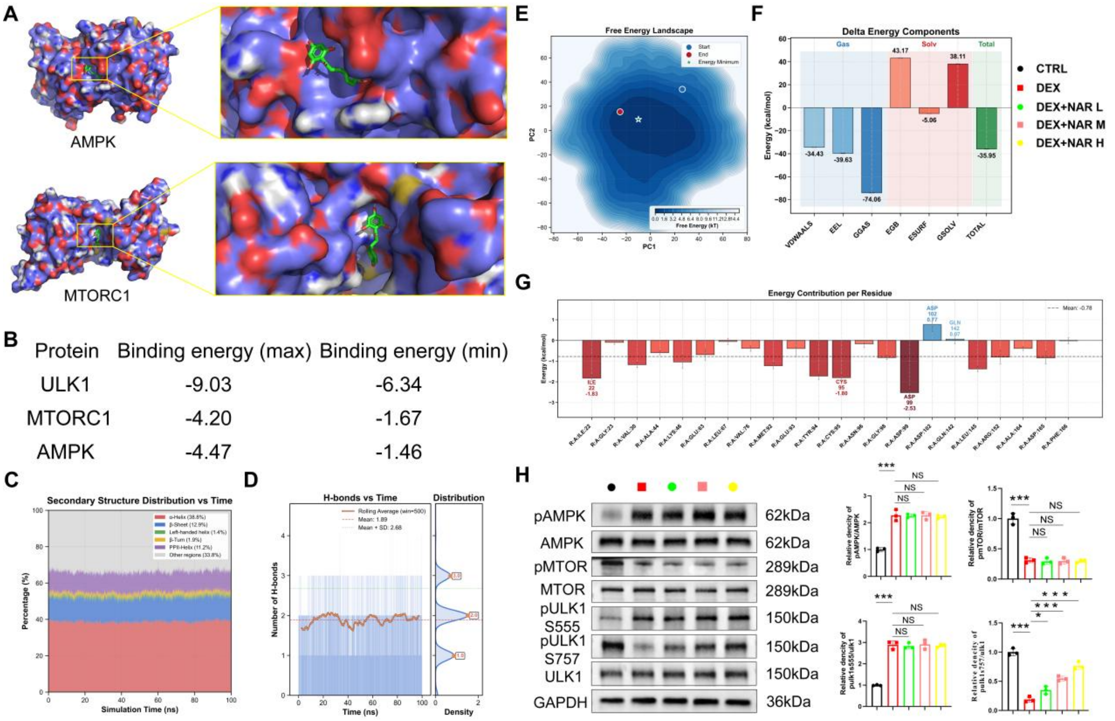

Figure S7. (**A**) Molecular docking image of NAR with AMPK and MTOR. (**B**) Molecular docking binding capacity of NAR to ULK1, MTOR and AMPK. (**C**) ULK1 secondary structure content over time. (**D**) ULK1-NAR hydrogen bond number plotted over time. (**E**) Free energy landscape map. (**F**) Analysis of the binding free energy composition of ULK1-NAR. (**G**) Residue energy contribution map of ULK1-NAR. (**H**) The levels of AMPK/MTOR/ULK1 signalling pathway-related proteins in BMSCs treated with 1 µM DEX and different concentrations of NAR (n = 3). Data are presented as mean ± SD. NS no significance, *$P < 0.05$, and ***$P < 0.001$ (vs. 1 µM DEX).

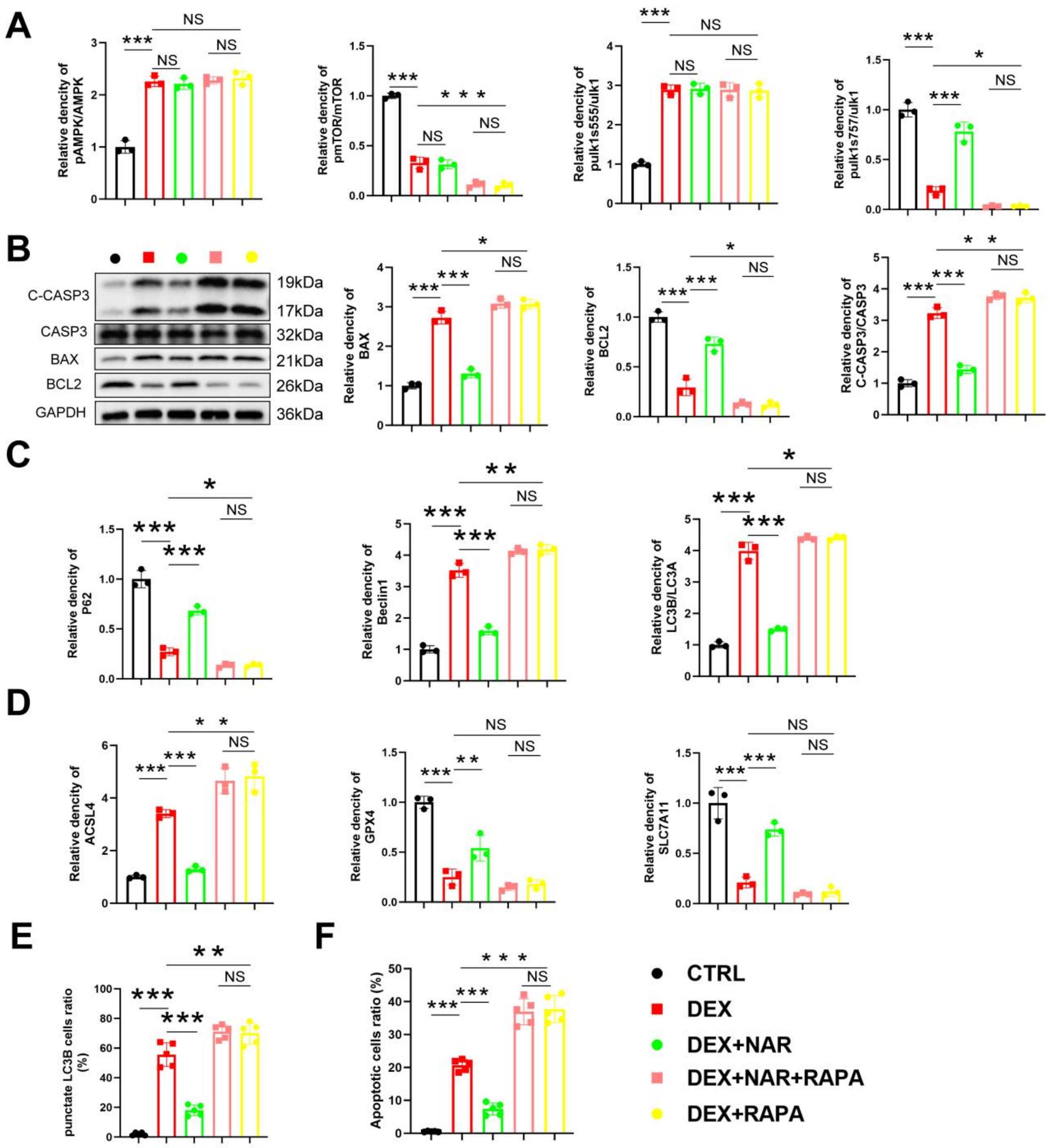

Figure S8. (**A**) The levels of AMPK/MTOR/ULK1 signalling pathway-related proteins in BMSCs treated with 1 μM DEX and 20 μM NAR and RAPA (n = 3). (**B**) Protein levels of C-CASP3, CASP3, BAX and BCL2 in 1 μM DEX, 3-MA or RAPA-treated BMSCs (n = 3). (**C-D**) Protein levels of P62, Beclin1, LC3B, ASCL4, GPX4 and SLC7A11 in BMSCs treated with 1 μM DEX, 20 μM NAR and RAPA (n = 3). (**E**) Quantitative analysis of mCherry-GFP-LC3B in BMSCs treated with 1 μM DEX, 20 μM NAR and RAPA (n = 5). (**F**) Apoptotic ratio in BMSCs treated with 1

μM DEX, 20 μM NAR and RAPA (n = 5). Data are presented as mean ± SD. NS no significance, *P < 0.05, **P < 0.01, and ***P < 0.001 (vs. 1 μM DEX or DEX+RAPA).

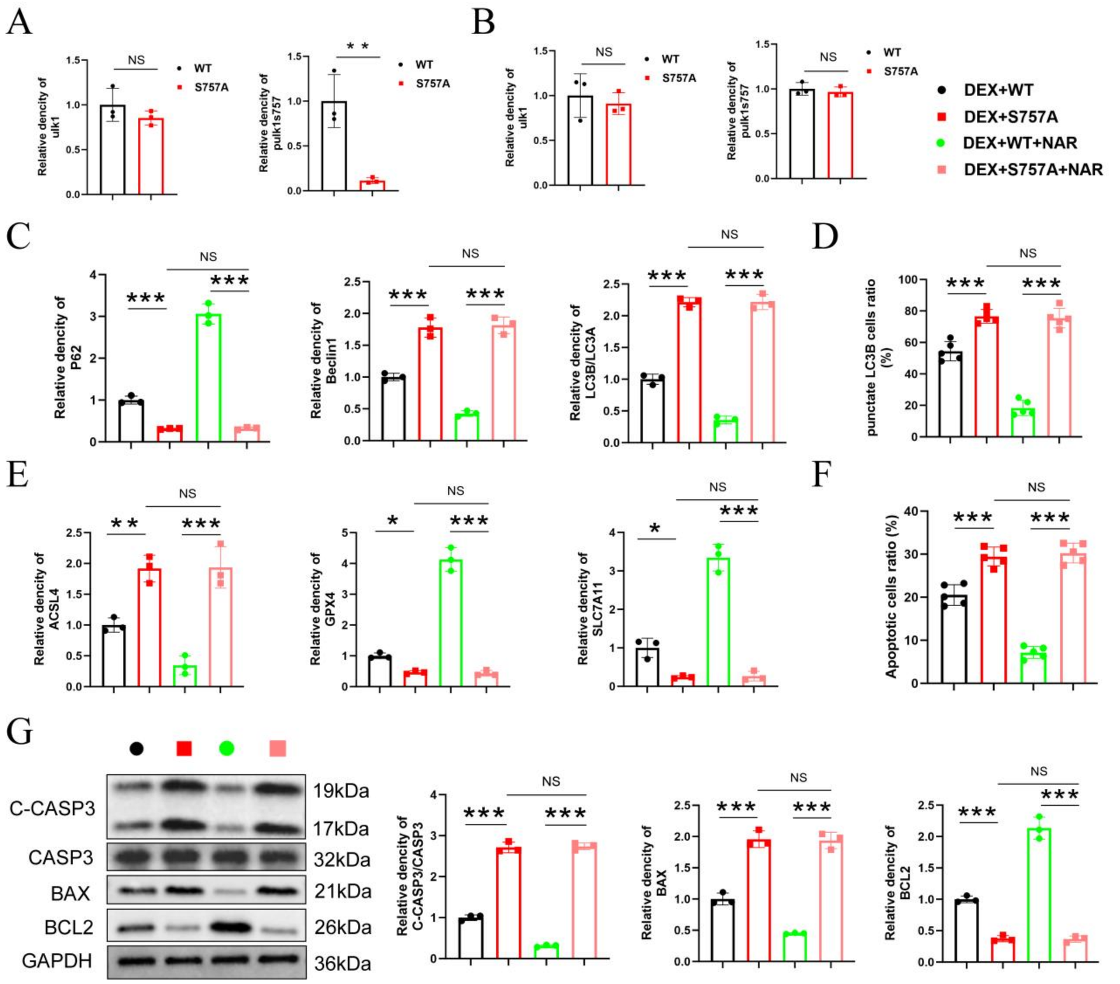

Figure S9. (**A-B**) Protein levels of ULK1 and pULK1 ser757 in BMSCs treated with S757A and NAR (n = 3). (**C**) Protein levels of P62, Beclin1 and LC3B in BMSCs treated with 1 μM DEX and WT or S757A or 20 μM NAR (n = 3). (**D**) Quantitative analysis of mCherry-GFP-LC3B in BMSCs treated with 1 μM DEX and WT or S757A or 20 μM NAR (n = 5). (**E**) Protein levels of ASCL4, GPX4 and SLC7A11 in BMSCs treated with 1 μM DEX and WT or S757A or 20 μM NAR (n = 3). (**F**) Apoptotic ratio in BMSCs treated with 1 μM DEX and WT or S757A or 20 μM NAR (n = 5). (**G**) Protein levels of C-CASP3, CASP3, BAX and BCL2 in BMSCs treated with 1 μM DEX and WT or S757A or 20 μM NAR (n = 3). Data are presented as mean ± SD. NS no significance, *P < 0.05, and ***P < 0.001 (vs. DEX+S757A or

DEX+S757A+NAR).

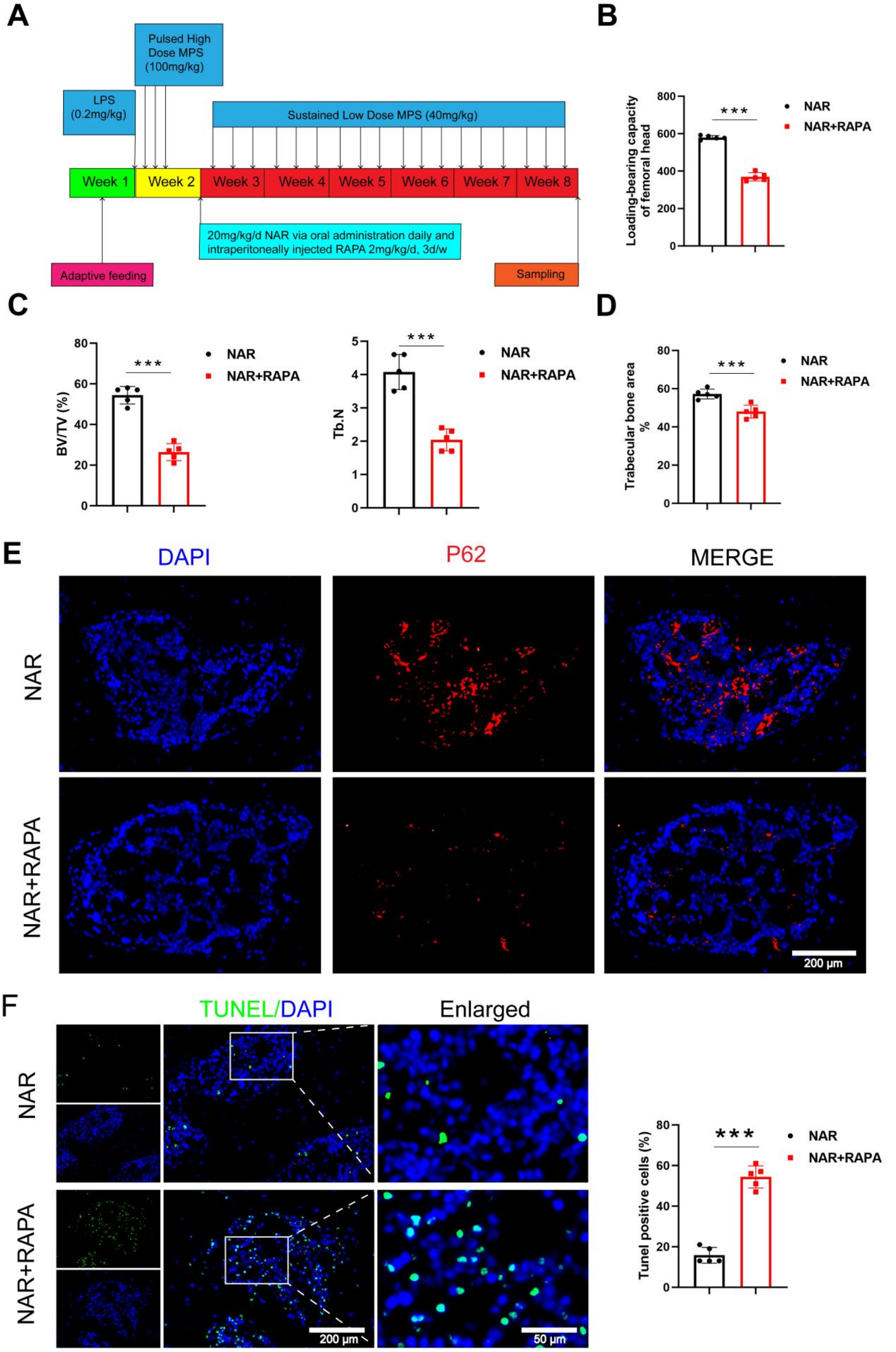

Figure S10. (**A**) The schematic diagram of the in vivo modelling procedure. (**B**) The

load-bearing capacity of rat femoral head samples (n = 5). (**C**) Quantitative analysis of BV/TV and Tb.N of rat femoral head samples (n = 5). (D) Quantitative analysis of trabecular bone area of rat femoral head samples (n = 5). (**E**) Representative IF staining images of P62 in rat femoral head samples. (**F**) Representative TUNEL staining images and quantitative analysis of rat femoral head samples (n = 5). Data are presented as mean ± SD. ***P < 0.001 (vs. NAR).

Table S1. Antibodies information

Antibodies	Company	Catalog #	Application/Dilution
CD90	Proteintech	66766-1-Ig	IF (1:100)
LC3B	Novus	NB100-2220	IF (1:100); WB (1:1000)
P62	Huabio	R1309-8	IF (1:100); WB (1:1000)
Beclin1	Abclonal	A7353	WB (1:1000)
C-CASP3	Cell Signaling Technology	9664S	WB (1:1000)
CASP3	Huabio	ET1608-64	WB (1:1000)
BAX	Huabio	ET1603-34	WB (1:1000)
BCL2	Huabio	ET1702-53	WB (1:1000)
GPX4	Abclone	A25009	IF (1:200); WB (1:1000)
ACSL4	Abclone	A22901	WB (1:10000)
SLC7A11	Novus	NB300-318	WB (1:1000)
ALP	Arigo	ARG57422	IF (1:100)
pULK1S757	Affinity	AF4387	IF (1:100)
pAMPK	Cell Signaling Technology	2535S	WB (1:1000)
AMPK	Cell Signaling Technology	5832S	WB (1:1000)
pMTOR	Cell Signaling Technology	5536T	WB (1:1000)
MTOR	Cell Signaling Technology	2983S	WB (1:1000)
pULK1S757	Cell Signaling Technology	6888S	WB (1:1000)
pULK1S555	Novus	NBP3-13124	WB (1:1000)
ULK1	Cell Signaling Technology	8054S	WB (1:1000)
GAPDH	Huabio	ET1601-4	WB (1:5000)
Goat anti-Mouse IgG (HRP-conjuagted)	Proteintech	SA00001-1	WB (1:5000)
Goat anti-Rabbit IgG (HRP-conjuagted)	Proteintech	SA00001-2	WB (1:5000)
Anti-Rabbit IgG for IHC	ZSGB-BIO	PV-6001	IHC (1:1000)
Anti-Mouse IgG for IF	Thermo Fisher Scientific	A11001	IF (1:1000)

Anti-Rabbit IgG for IF	Thermo Fisher Scientific	A31572	IF (1:1000)

Table S2. Sequences of primers

Primer name	Sequence
RUNX2 forward	5′-GAGGGCACAAGTTCTATCTGGA-3′
RUNX2 reverse	5′-GGTGGTCCGCGATGATCTC-3′
OSX forward	5’-CCCTTCTCAAGCACCAATGG-3’
OSX reverse	5’-AAGGGTGGGTAGTCATTTGCATA-3’
ALP forward	5′-GTTGGGGGTGCCCACGGT-3′
ALP reverse	5′-CCTTGGACAGAGCCATGTATG-3′
β-Actin forward	5′-GGAGATTACTGCCCTGGCTCCTA-3′
β-Actin reverse	5′-GACTCATCGTACTCCTGCTTGCTG-3′
